# Comparative study of gut microbiota in wild and captive Malaysian Mahseer (*Tor tambroides*)

**DOI:** 10.1002/mbo3.734

**Published:** 2018-10-23

**Authors:** Chun K. Tan, Ikhsan Natrah, Iswan B. Suyub, Marilyn J. Edward, Nazrien Kaman, Anjas A. Samsudin

**Affiliations:** ^1^ Agro‐Biotechnology Institute (ABI) National Institutes of Biotechnology Malaysia (NIBM) Serdang Malaysia; ^2^ Faculty of Agriculture Department of Aquaculture Universiti Putra Malaysia (UPM) Serdang Malaysia; ^3^ Faculty of Agriculture Department of Animal Science Universiti Putra Malaysia (UPM) Serdang Malaysia

**Keywords:** 16S rDNA, gut microbiota, Illumina MiSeq, metagenetic sequencing, next‐generation sequencing, *Tor tambroides*

## Abstract

**Aims:**

The aim of this study was to identify and compare the gut microbial community of wild and captive *Tor tambroides* through 16S rDNA metagenetic sequencing followed by functions prediction.

**Methods and results:**

The library of 16S rDNA V3‐V4 hypervariable regions of gut microbiota was amplified and sequenced using Illumina MiSeq. The sequencing data were analyzed using Quantitative Insights into Microbial Ecology (QIIME) pipeline and Phylogenetic Investigation of Communities by Reconstruction of Unobserved States (PICRUSt). The most abundant bacterial phyla in both wild and captive *T. tambroides* were Firmicutes, Proteobacteria, Fusobacteria and Bacteroidetes. *Cetobacterium* spp., Peptostreptococcaceae family, *Bacteroides* spp., Phosphate solubilizing bacteria *PSB‐M‐3,* and *Vibrio* spp. were five most abundant OTU in wild *T. tambroides* as compared to *Cetobacterium* spp., *Citrobacter* spp., Aeromonadaceae family, Peptostreptococcaceae family and *Turicibacter* spp. in captive *T. tambroides*.

**Conclusion:**

In this study, the specimens of the wild *T. tambroides* contain more diverse gut microbiota than of the captive ones. The results suggested that *Cetobacterium* spp. is one of the core microbiota in guts of *T. tambroides*. Besides, high abundant *Bacteroides* spp., *Citrobacter* spp., *Turicibacter* spp., and *Bacillus* spp. may provide important functions in *T. tambroides* guts.

**Significance and impact of the study:**

The results of this study provide significant information of *T. tambroides* gut microbiota for further understanding of their physiological functions including growth and disease resistance.

## 1 INTRODUCTION

Fish of the genus *Tor*, commonly known as mahseers, are important to most nations in the Asian region due to its biodiversity and high‐value (Ng, 2004). *Tor tambroides* also known as “empurau” in Sarawak or “kelah merah” in Peninsular Malaysia is the most valued freshwater fish species in Malaysia (Ingram, Sungan, Tinggi, Sim, & De Silva, 2007). The *T. tambroides* has generated much interest in its artificial propagation for both conservation and aquaculture production due to its high market demand, high flesh quality and high commercial value (Ng, Abdullah, & De Silva, 2008).

One of the major problems with *T. tambroides* captive breeding is the slow growth of *T. tambroides* (Lee et al., 2014). There were many studies on the different feed formulations and feed additives that improved the growth rate of *T. tambroides* (Ishak, Kamarudin, Ramezani‐Fard, & Yusof, 2016; Kamarudin, Ramezani‐Fard, Saad, & Harmin, 2011; Misieng, Kamarudin, & Musa, 2011; Ng & Andin, 2011; Ng et al., 2008; Ramezani‐Fard, Kamarudin, Saad, Harmin, & Goh, 2012). There were also studies on effects of host gut‐derived probiotic bacteria to *T. tambroides* where the probiotic improved nutrients utilization and metabolism, adjusting gut microbiota balance and enhanced growth by promoting muscle fiber hypertrophy (Asaduzzaman, Iehata, et al., 2018; Asaduzzaman, Sofia, et al., 2018). Nevertheless, there is no report on the phylogenetic and functional characterization of gut microbiota of *T. tambroides*.

Gut microbiota can be considered as an “extra organ” due to its crucial role in intestinal development, homeostasis and immunological protection, growth and health (O'Hara & Shanahan, 2006). The gut microbiota in vertebrate is complex and contains diverse and abundant bacteria, archaea, viruses, and fungi (Liu et al., 2015; Neuman & Koren, 2015). Gut microbiota of aquatic animals is transient and has higher fluidity than terrestrial animals; thus, changes in environmental factors such as temperature, salinity, trophic level, and host phylogeny may affect the gut microbial community (Denev, Staykov, Moutafchieva, & Beev, 2009; Guerreiro et al., 2016; Ringø et al., 2016; Sullam et al., 2012). More than 99% of environmental prokaryotes including the gut microbiota of animals are unculturable in laboratory that limits our understanding of microbial physiology, genetics, and community ecology (Schloss & Handelsman, 2005). The development of next‐generation sequencing (NGS) technology allows the recognition of discrete populations (culturable and unculturable) based on DNA sequences in the environmental samples (Konstantinidis & Rosselló‐Móra, 2015; Tarnecki, Burgos, Ray, & Arias, 2017). Esposito and Kirschberg (2014) clarified that the metagenomic study means the whole genome sequencing and analysis of each member of the microbial community in an environmental sample by 16S rDNA‐based sequencing should be called as metagenetic sequencing.

Illumina MiSeq (Illumina, USA) has been widely used for 16S rRNA gene sequencing of gastrointestinal tract microbiota of freshwater fishes such as blunt snout, grass carp, mandarin fish, topmouth cutler, common carp, crucian carp, silver carp, bighead carp, and Prussian carp (Kashinskaya et al., 2015; Liu et al., 2015) and marine fishes such as emerald rockcod, crocodile icefish, ploughfish, bald rockcod, yellowtail scad, brown‐marbled grouper, spotted coral grouper and Atlantic salmon (Dehler, Secombes, & Martin, 2017; Hennersdorf et al., 2016; Song et al., 2016).

The objectives of this study were to identify and compare gut microbiota in wild and captive *T. tambroides*. Determination of core bacteria and prediction of their functions in gut microflora lead to identification of potential bacteria that could be used as probiotics to improve growth performance and disease resistance of *T. tambroides* in captivity.

## 2 MATERIALS AND METHODS

### 2.1 Fish sampling and species verification

Three captive adult *T. tambroides* (standard length 35.77 ± 1.39 cm, weight 960.57 ± 58.29 g) were obtained from hatchery at Agro‐Biotechnology Institute (ABI) on 6 April 2015. The captive fish were obtained from the wild and reared in hatchery for 3 years. They were fed twice daily (8.00 a.m. and 4.00 p.m.) with commercial floating pellet containing 42% crude protein and 6% lipid. Fish were reared in rectangular fiberglass tank with 1,500 L of dechlorinated tap water with continuous aeration. Each tank was attached to a recirculating aquaculture system (RAS) with 30% water changes fortnightly. Three wild adult *T. tambroides* (standard length 31.73 ± 0.78 cm, weight 630.27 ± 56.32 g) were obtained by angling from Kenyir Lake, Terengganu, Malaysia (GPS Coordinates: 5°0′14″N, 102°38′19″E) on 13 April 2015. These fish were packed in Kenyir Lake water and transported alive to ABI, Serdang, Selangor, Malaysia (GPS Coordinates: 2°59′18″N, 101°41′52″E) (approx. 5 hr). These fish were processed upon arrived at the destination.

DNA of the fish was extracted from dorsal fin samples using Phenol‐Chloroform‐Isoamyl‐Alcohol (PCI) DNA extraction method (Tan et al., 2008). The cytochrome b gene was amplified using GluDG‐L (5′‐TGACTTGAARAACCAYCGTTG‐3′) and CB2‐H (5′‐CCCTCAGAATGATATTTGTCCTCA‐3′) primers (Palumbi et al., 2002). Reaction mixture (25 μl) included HotStarTaq Plus Master Mix (10 μl) (Qiagen, Germany), forward and reverse primers (1 μM and 5 μl of each) and template DNA (10 ng). Amplification conditions were the following: initial denaturation at 95°C for 5 min; denaturation at 95°C for 45 s, annealing at 47°C for 45 s, elongation at 72°C for 45 s (25 cycles); final elongation at 72°C for 7 min. The PCR products were purified using QIAquick PCR Purification Kit (Qiagen, Germany). DNA sequencing was outsourced to First BASE Laboratories Sdn. Bhd. (Malaysia). The results were analyzed using NCBI BLASTn (NCBI, 1988).

### 2.2 Fish dissection and DNA extraction

*Tor tambroides* were anesthetized using 30 ppm clove oil (Neiffer & Stamper, 2009) and euthanized by pithing (Leary et al., 2013). Fish skin was disinfected with 70% ethanol prior to autopsy. The abdomen of fish was dissected using sterile instruments in laminar flow cabinet. The gut samples were removed and separated from other internal organs. The gut parts from esophagus to anus were then cut into small pieces and placed in sterile phosphate‐buffered saline (PBS) (Nie, Zhou, Qiao, & Chen, 2017) followed by mixing with vortex and kept at −80°C. Gut microbiota DNA in these gut samples was extracted using PCI DNA extraction method (Tan et al., 2008).

### 2.3 Library preparation and MiSeq sequencing

V3–V4 hypervariable regions of 16S rRNA genes of gut microbiota were amplified by polymerase chain reaction (PCR) using primers (Forward primer: 5′‐TCGTCGGCAGCGTCAGATGTGTATAAGAGACAGCCTACGGGNGGCWGCAG‐3′ and Reverse primer: 5′GTCTCGTGGGCTCGGAGATGTGTATAAGAGACAGGACTACHVGGGTATCTAATCC‐3′) according to the manufacturer's instructions. Reaction mixture (25 μl) included 2X KAPA HiFi HotStart ReadyMix (12.5 μl) (Kapa Biosystems, USA), forward and reverse primers (1 μM and 5 μl of each), and template DNA (7.5 ng). Amplification conditions were as the following: initial denaturation at 95°C for 3 min; denaturation at 95°C for 30 s, annealing at 55°C for 30 s, elongation at 72°C for 30 s (25 cycles); final elongation at 72°C for 5 min.

Amplicons were cleaned up followed by PCR to attach unique index adapter pairs to the amplicons using Nextera XT Index kit (Illumina). These indexed DNA libraries were cleaned up with Agencourt AMPure XP (Beckman Coulter, USA) followed by concentration quantification using Qubit dsDNA HS Assay Kit and Qubit 2.0 Fluorometer (Thermo Fisher Scientific, USA) and size validation using Agilent DNA 1000 Kit and Agilent 2100 Bioanalyzer (Agilent, USA).

The libraries were serial diluted and quantified by quantitative real‐time PCR (qRT‐PCR) through Eppendorf Mastercycler RealPlex^2^ (Eppendorf, Germany) followed by normalization to 4 nM and pooled into one tube. Pooled DNA libraries were denatured and spiked with 15% denatured PhiX as quality control. A mixture of 600 μl of denatured pooled DNA libraries with denatured PhiX loaded into the sample well in MiSeq Reagent Kit v2 (2 × 250 cycles) (Illumina) and sequenced using Illumina MiSeq (Illumina) at Malaysia Genome Institute (MGI), Kajang, Selangor, Malaysia. All sequences were also submitted to NCBI Sequence Read Archive (SRA).

### 2.4 Data analysis using Quantitative Insights into Microbial Ecology (QIIME)

The analysis of MiSeq sequencing results was done using Quantitative Insights into Microbial Ecology (QIIME ver. 1.9.0) pipeline (Caporaso, Kuczynski, et al., 2010). Adapter sequences were trimmed from the paired‐end forward and reverse reads and merged. Merged reads were quality filtered at Phred Quality Score of 20 (Q20) (Cock, Fields, Goto, Heuer, & Rice, 2010). Length filter was used to remove reads shorter than 100 bp (below 20% of the library length) to avoid unspecific match that will disturb the accuracy of the calling (Edgar, 2010). Chimeric sequences were removed using RDP Gold databases as reference (Edgar, Haas, Clemente, Quince, & Knight, 2011). De novo OTU picking strategy was used as it did not cause any information lost although may be time‐consuming for large datasets (Edgar, 2010; Edgar et al., 2011).

The OTUs in generated OTU BIOM file were summarized into different taxonomic levels. Taxa summary plots were plotted to show the differences in taxonomic levels of the samples. Alpha rarefactions curves were plotted to determine the adequacy of sequencing depth. Alpha diversity indexes (Chao1, Shannon and Simpson) were calculated to explain the species richness and diversity in each sample (Udayangani et al., 2017). Good's Coverage estimator was used to estimate the percentage of the total species that are represented in a sample. In beta diversity analysis, the number of sequences per sample had been rarified to equal number based on the sample which had the lowest sequences number. Principle coordinates analysis (PCoA) was used to visualize similarities or dissimilarities of data based on phylogenetic or count‐based distance metrics. Weighted UniFrac was used in the PCoA analysis of this study because it accounted for differences in relative abundances of each taxon within the communities (Lozupone, Hamady, Kelley, & Knight, 2007). Mann–Whitney *U* test was used to determine the differences of the gut microbial communities in the wild and captive *T. tambroides* (Jonsson, Österlund, Nerman, & Kristiansson, 2016).

### 2.5 Functions prediction using Phylogenetic Investigation of Communities by Reconstruction of Unobserved States (PICRUSt)

PICRUSt (ver. 1.1.0) was used to predict the metabolic functions of the microbial communities in each sample (Langille et al., 2013). Closed reference OTU picking strategy was used with the GreenGenes database (version 13.5) as reference at 97% identity threshold (Caporaso, Bittinger, et al., 2010; DeSantis et al., 2006). The OTU table was then normalized, and the microbiota functions were predicted with referenced to Kyoto Encyclopedia of Genes and Genomes (KEGG) Orthology (KO) database (Kanehisa & Goto, 2000).

## 3 RESULTS

### 3.1 Dissection and species verification

Autopsy of *T. tambroides* revealed that the gut digesta color of wild *T. tambroides* obtained from Kenyir Lake was green while it was brown in captive *T. tambroides* obtained from hatchery which was fed with commercial floating feed pellets. The cytochrome b gene of both wild and captive *T. tambroides* was analyzed using NCBI BLASTn and found to be 98%–99% similar to cytochrome b gene in complete mitochondrial genome of *T. tambroides* (GenBank Accession Number: JX444718.1) (National Center for Biotechnology Information (NCBI), 1988; Norfatimah, Teh, Salleh, Mat Isa, & SitiAzizah, 2014).

### 3.2 Metagenetic sequencing of wild and captive *T. tambroides* gut microbiota with QIIME analysis

The de novo OTU picking generated 7,749 and 9,468 OTUs for wild and captive *T. tambroides* gut microbiota, respectively (Table 1). Nevertheless, the number of species found in wild *T. tambroides* was 501 as compared to 442 in captive ones. 304 genera were shared between wild and captive *T. tambroides* (Supporting Information Table SA). All sequencing data (three wild and three captive *T. tambroides* gut microbiome) were submitted to NCBI Sequence Read Archive (SRA) under accession number of SRP094031.

**Table 1:** Summary of gut microbiome in wild and captive *Tor tambroides*

Sample	Sequences after merging of forward and reverse reads	Sequences after QC quality filter	Sequences after length filter (>100 bp)	Sequences after chimera filter	Operational taxonomic units (OTUs) after OTU picking
Wild	785,128	751,324	751,318	558,171	7,749
Captive	1,024,668	983,240	983,010	702,194	9,468

Each value was a mean value calculated from the raw data.

### 3.3 Alpha diversity analysis of *T. tambroides* gut microbiota

**Figure 1:**
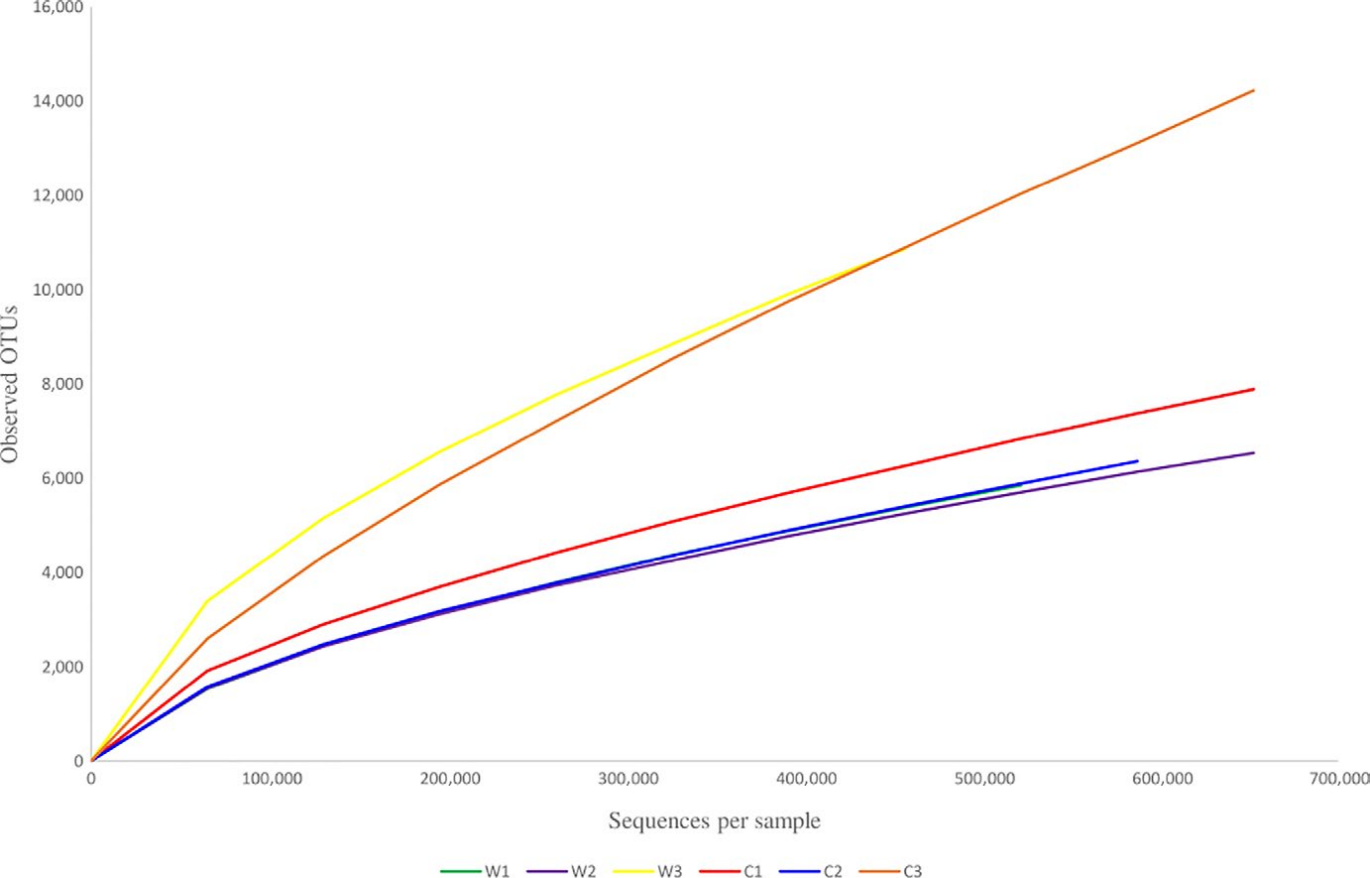
Alpha rarefaction curves of wild and captive *Tor tambroides* gut microbiota (C1–C3: biological replicates of captive *T. tambroides*; W1–W3: biological replicates of wild *T. tambroides*)

The OTUs found in both wild and captive *T. tambroides* were reduced as the number of sequences increased at 50,000 sequences per sample (Figure 1). In Table 2, Good's Coverage confirmed that the sequencing covered up to 99% of all gut microbiota in wild and captive *T. tambroides*. Chao1 index showed that captive *T. tambroides* gut microbiota had higher species richness than wild *T. tambroides*. Nevertheless, both Shannon and Simpson indexes for wild *T. tambroides* gut microbiota were higher than captive *T. tambroides*, indicated higher species diversity in fish that live in natural environment.

**Table 2:** Summary of alpha diversity of wild and captive *Tor tambroides* gut microbiota

Sample	Chao1	Shannon	Simpson	Good's coverage
Wild	19,280.31	4.83	0.87	0.99
Captive	26,814.36	4.44	0.81	0.99

### 3.4 Gut taxonomy of wild and captive *T. tambroides*

**Figure 2:**
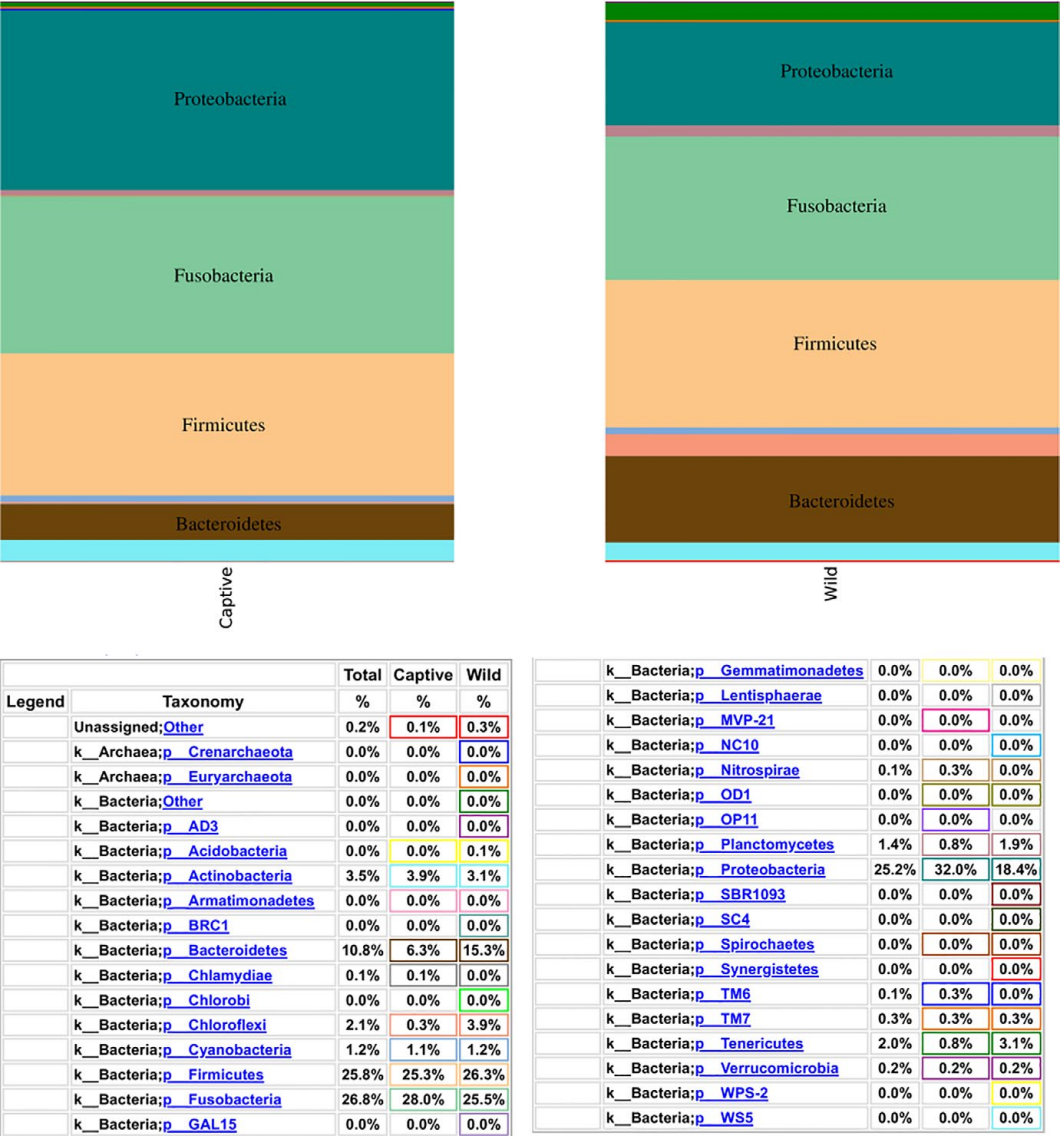
Relative abundance of phyla found in wild and captive *Tor tambroides* gut microbiome

The gut microbiota of wild *T. tambroides* was dominated mainly by Firmicutes followed by Fusobacteria, Proteobacteria, and Bacteroidetes which together accounted for 85.7% of total population. Gut of captive *T. tambroides* was dominated mainly by Proteobacteria followed by Fusobacteria, Firmicutes and Bacteroidetes which together accounted for 91.67% of total population (Figure 2). The most abundant genus in wild *T. tambroides* gut microbiota was *Cetobacterium* (23.48%), followed by genera in Peptostreptococcaceae family (11.87%), *Bacteroides* (9.60%), *PSB‐M‐3* from Erysipelotrichaceae family (7.70%), *Vibrio* (4.94%), and others (42.41%). *Cetobacterium* (29.07%) also was the most abundant genus in captive *T. tambroides* gut microbiota, followed by *Citrobacter* (9.35%), genera in Aeromonadaceae family (8.63%), genera in Peptostreptococcaceae family (7.66%), *Turicibacter* (6.47%), and others (38.82%) (Supporting Information Table SA). The 10 most abundant unique species in either wild or captive samples were listed in Table 3. These unique species only existed in small percentages (<0.31%).

**Table 3:** Ten most abundant unique OTUs in either wild or captive *Tor tambroides* gut

Wild *T. tambroides*	Percentage (%)	Captive *T. tambroides*	Percentage (%)
g__*Synechococcus*	0.306	g__*Virgibacillus*	0.200
o__SHA‐20;f__;g__	0.185	g__*Geobacillus*	0.049
c__Betaproteobacteria; Other; Other; Other	0.107	c__OP11‐4;o__;f__;g__	0.014
g__*Methylocaldum*	0.105	g__*Salinivibrio*	0.013
o__HOC36;f__;g__	0.099	g__*Pseudoxanthomonas*	0.012
c__Betaproteobacteria;o__;f__;g__	0.080	g__*Jeotgalicoccus*	0.011
o__ASSO‐13;f__;g__	0.077	g__*Cloacibacterium*	0.008
f__A4b;g__	0.075	g__*Ureibacillus*	0.007
f__Methylocystaceae; Other	0.061	f__[Tissierellaceae];Other	0.005
f__Pseudanabaenaceae; Other	0.045	g__*Tepidimicrobium*	0.004

### 3.5 Beta diversity analysis of wild and captive *T. tambroides* gut microbiota

**Figure 3:**
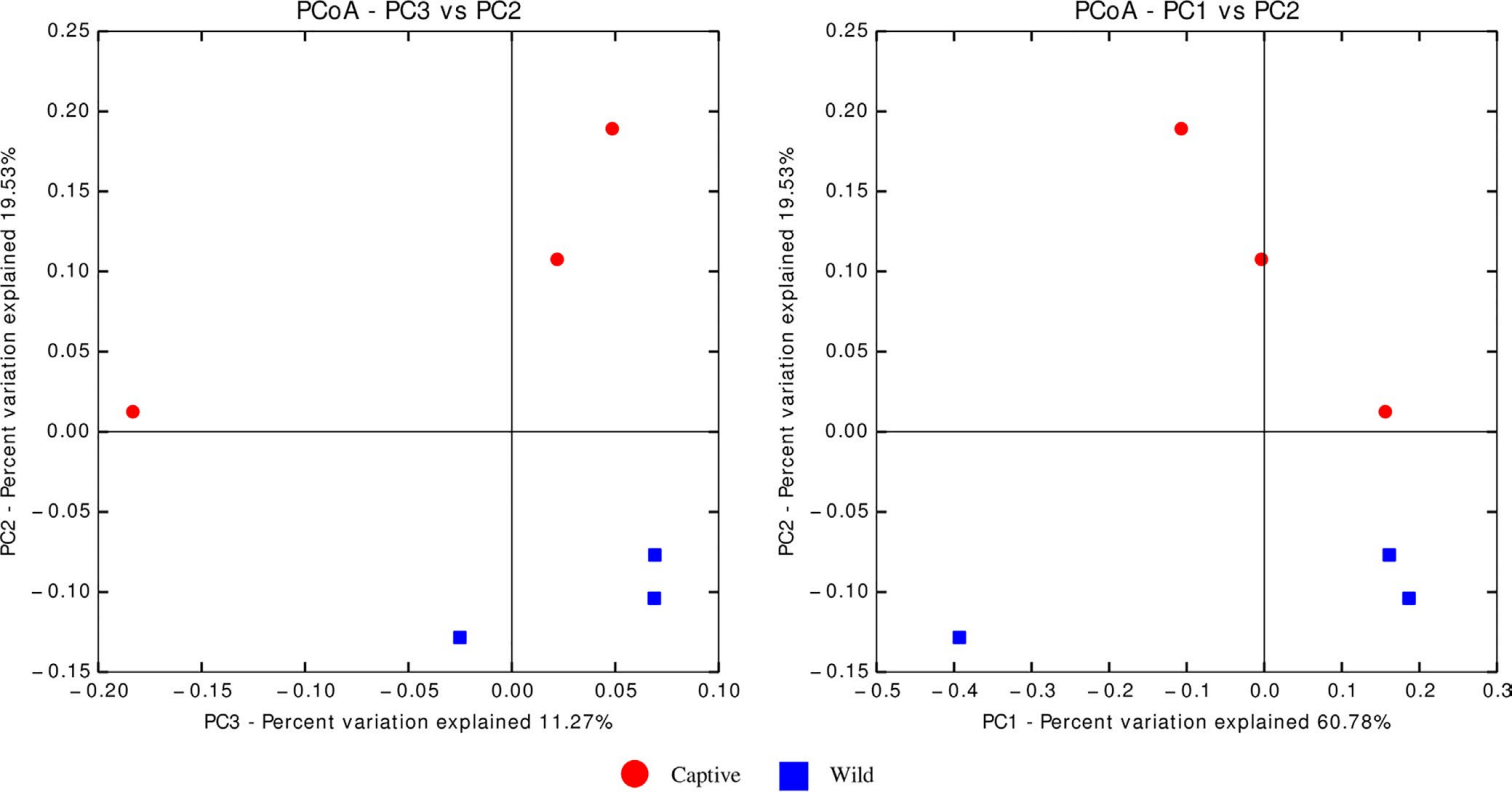
Principal Coordinates Analysis (PCoA) plots of beta diversity analysis based on weighted UniFrac distance metric

**Figure 4:**
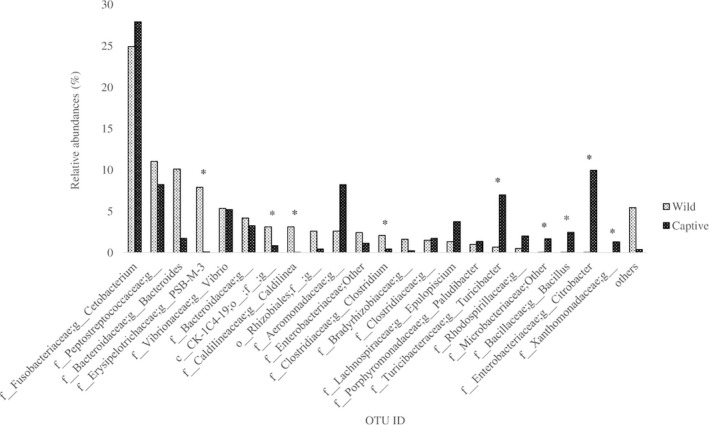
Comparison of top 15 most abundant observed bacteria in wild and captive *Tor tambroides* guts (Bars with * indicated significant differences between wild and captive *T. tambroides* samples)

The PCoA plots in Figure 3 showed clusters based on wild and captive samples were observed at Principal Coordinate 1 versus Principal Coordinate 2 (PC1 vs PC2) and PC3 vs PC2. *Cetobacterium* spp. was the highest OTU found in both wild and captive *T. tambroides* gut microbiota but there was no any significant difference between both samples (Figure 4). Unclassified species from genus *PSB‐M‐3*, unclassified order from class CK‐1C4‐19, *Caldilinea* spp., and *Clostridium* spp. were significantly higher (*p *<* *0.05) in wild *T. tambroides*. On the other hand, *Turicibacter* spp., unclassified genus from Rhodospirillaceae family, unclassified genus from Microbacteriaceae family, *Bacillus* spp., *Citrobacter* spp., and unclassified genus from Xanthomonadaceae family were significantly higher (*p *<* *0.05) in captive *T. tambroides*.

### 3.6 Predicted metabolic functions using PICRUSt

**Figure 5:**
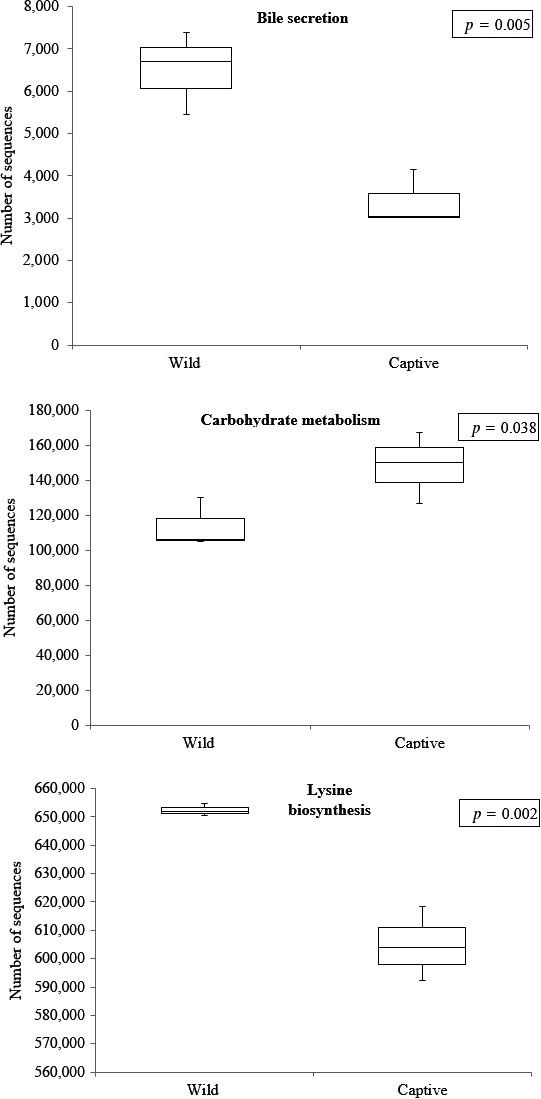
Box‐plots showed three significant predictive functions (bile secretion, carbohydrate metabolism and lysine biosynthesis) of gut microbial communities in wild and captive *Tor tambroides*

PICRUSt analyses revealed a total of 293 predicted functions where 277 functions existed in both samples (Supporting Information Table SB). 10 unique functions were found only in wild *T. tambroides* gut microbiota while six unique functions were only found in captive *T. tambroides* gut microbiota. Bile secretion and lysine biosynthesis were significantly higher (*p *<* *0.05) in wild *T. tambroides* gut microbiota while carbohydrate metabolism was significantly higher (*p *<* *0.05) in captive *T. tambroides* (Figure 5).

## 4 DISCUSSION

Although previous studies (Esa et al., 2008; Sati et al., 2013) mostly used cytochrome c oxidase subunit I (COI) for mahseer species identification, all six *T. tambroides* used in this study for metagenetic analysis were identified using mitochondrial Cytochrome b (CytB) gene. Comparison of CytB gene and COI gene showed that CytB gene is more accurate to construct phylogeny trees and reveal evolutionary relationships, and it gave better resolution during separating species based on sequence data (Tobe, Kitchener, & Linacre, 2011). Although Hampala showed considerable geographical variation in coloration and morphological characteristics, the mitochondrial cytochrome b gene sequencing was able to resolve phylogenetic relationship of Hampala fishes (Ryan & Esa, 2006). It was necessary to accurately verify the species of the mahseer fish used in this study prior to MiSeq sequencing since other fishes such as *Tor* spp. and *Neolissochilus* spp. are morphological similar to *T. tambroides* (Laskar et al., 2013). Identification of fish species based on morphological appearances is also subjective and can lead to misidentification.

Due to the different types of the feeds, eating habits and habitats of the wild and captive *T. tambroides*, it is anticipated that their gut microbiota community will be different (Li et al., 2014; Ringø et al., 2016). The wild *T. tambroides* in Kenyir Lake lives in natural environment that contains various types of algae belonging to cyanophytes, bacillariophytes and chlorophytes (Rouf, Phang, & Ambak, 2010). Digesta of all three wild *T. tambroides* were green in color indicating these fish may be fed in various kinds of algae or plant as food in Kenyir Lake. In contrast, the gut digesta of captive *T. tambroides* was brown due to the formulated pellet diet which consists of complete nutrients from animal and plant sources. Therefore, *T. tambroides* in captivity would grow faster than wild *T. tambroides* at the same age. Temperature fluctuation had effect to the composition of the gut microbiota in farmed Atlantic salmon (Neuman et al., 2016). Various habitats with different environmental factors formed the gut microbiota composition of Atlantic salmon parr (Dehler et al., 2017). Higher species richness of gut microbiota was found in Atlantic salmon parr exposed to open water natural environment than in captive reared ones (Dehler et al., 2017). Although the weight and sizes of captive *T. tambroides* were higher compared to wild *T. tambroides* used in this study, it was anticipated that their gut microbiota will be highly influenced by the living environment, feed, and feeding habits.

Operational taxonomic units (OTUs) are groups of sequences that clustered together based on percent similarity threshold (typically 97%) assuming they delineate a species (Nguyen, Warnow, Pop, & White, 2016). Number of OTUs found in wild *T. tambroides* guts were lower than captive *T. tambroides*. However, one OTU does not represent one species because OTUs were clustered based on similarity of the other sequences in the bacterial community regardless of whether the sequence is represented by references within a taxonomy outline (Schloss & Westcott, 2011). Thus, a few OTUs may refer to the same species. In alpha diversity analysis, high Chao1 value indicated high species richness (Hughes, Hellmann, Ricketts, & Bohannan, 2001). In our study, the Shannon and Simpson indexes of the wild *T. tambroides* gut microbiota were higher indicating higher bacterial diversity compared to captive *T. tambroides* gut microbiota. Shannon and Simpson indexes are calculated based on both species richness and species evenness of the microbial community (Gihring, Green, & Schadt, 2011; Spellerberg & Fedor, 2003).

Firmicutes and Bacteroidetes are able to degrade wide range of polysaccharides (Cockburn & Koropatkin, 2016). This may explain the higher percentages of Firmicutes and Bacteroidetes in wild *T. tambroides* gut microbiota as their habitat in Kenyir Lake contains huge amount of periphyton algae (Rouf et al., 2010). Cell wall of green algae contains various polysaccharides such as cellulose, pectins, hemicelluloses, lignin, and others (Domozych et al., 2012). Digesta in the wild *T. tambroides* guts used for this study appeared to be green in color indicated these fish consumed a lot of algae or plant as food which had cell walls made of polysaccharides. In PCoA plots, sample data points cluster together indicated high similarity of gut microbial population among the samples. One of the data of captive *T. tambroides* sample was not cluster close to the rest as captive *T. tambroides* samples that were obtained from separated tanks in hatchery where the different microbial community in the tank water could contribute to these dissimilarities. Bacterial communities in water affected Nile tilapia larvae gut microbial communities (Giatsis et al., 2015).

*Cetobacterium* spp. was the most abundant species without any significant differences in both wild and captive samples suggesting that it is a core species in *T. tambroides* guts. The colonization of this species in captive *T. tambroides* even after 3 years of rearing in hatchery condition may indicate their roles and functions in the fish gut. Anaerobic *Cetobacterium* spp. promotes decomposition of consumed organic debris, phytoplankton, or zooplankton (Borsodi et al., 2017). This species was also common in intestinal tracts of goldfish, common carp, grass carp, ayu, tilapia, zebrafish, rainbow trout, channel catfish, largemouth bass, and bluegill (Adeoye et al., 2016; Etyemez & Balcázar, 2015; Larsen, Mohammed, & Arias, 2014; Roeselers et al., 2011; Tsuchiya, Sakata, & Sugita, 2007; Van Kessel et al., 2011). However, the effects of this species have never been tested in fish. This may be due to the fact that *Cetobacterium* spp. is obligate anaerobe that will die under normal atmospheric condition thus hinder the possibility of using this species as probiotics in aquaculture production. There was a report stated that bacteria‐mediated cobalamin biosynthesis was supported by the presence of cobalamin synthesizers such as *Bacteroides*,* Lactobacillus,* and *Cetobacterium* (Koo et al., 2017). Besides, *C. somerae* was reported to produce vitamin B_12_ which also known as cobalamin (Tsuchiya et al., 2007).

The number of *Bacillus* spp. was higher in captive *T. tambroides* gut samples. The *Bacillus* spp. may originate from probiotics capsules that were added into the tanks few years ago. *Bacillus* species have been widely used as probiotics in aquaculture industry. *Bacillus* spp. was discovered to possess anti‐pathogenic properties such as antibacterial and anti‐quorum sensing properties (Chu, Zhou, Zhu, & Zhuang, 2014). *Bacillus licheniformis* and *Bacillus pumilus* showed antibacterial activity against *Aeromonas hydrophila* infection (Ramesh, Vinothkanna, Rai, & Vignesh, 2015; Shobharani, Padmaja, & Halami, 2015). *Bacillus subtilis* in diets increased growth rate of *T. tambroides*, upregulated immune‐related genes, and improved stress tolerance toward temperature changes (Nguyen, 2015). Spore production ability of *B. subtilis* has the potential to be used as delivery system for vaccine or recombinant spores that expressed surface enzyme which induced innate and adaptive immunity, systemic and local mucosal immunity (Jiang et al., 2017).

Dominance of *Citrobacter* genus was observed in captive *T. tambroides* gut microbiome. *Citrobacter freundii* had inhibitory effects against *A. hydrophila* (Aly, Ahmed, Ghareeb, & Mohamed, 2008). In contrast, *C. freundii* isolated from intestinal tract of farmed grass carp showed pathogenicity to mice and zebrafish (Lü et al., 2011). *Clostridium* spp. was higher in wild *T. tambroides* gut. *Clostridium butyricum* was reported as a potential probiotic that has strong adhesion and antagonistic activity against *A. hydrophila* and *Vibrio anguillarum* (Pan et al., 2008).

Bile is essential for digestion and absorption of fats and removal of excess cholesterol, bilirubin, drugs, and toxic compounds (Kanehisa, Tanabe, Sato, & Morishima, 2017). Gut microbiota is capable to convert bile acids into secondary bile acids which modulate its signaling properties that regulate diverse metabolic pathways in the host (Ramírez‐Pérez, Cruz‐Ramón, Chinchilla‐López, & Méndez‐Sánchez, 2017). *Eubacterium lentum* and *Clostridium perfringens* were reported to possess the capability to produce iso‐bile acids (Hirano & Masuda, 1981; Hirano, Masuda, Oda, & Mukai, 1981). Enzymes from gut microbiota may contribute significantly to bile acid metabolism and essential for bile acid homeostasis in the host and contributed to host health (Long, Gahan, & Joyce, 2017). Higher bile secretion functions of gut microbiota in wild *T. tambroides* may offer protection to the fishes that exposed to natural environment. In this study, the *Clostridium* spp. was 2.09% in wild samples as compared to 0.42% in captive ones.

Carbohydrate needed to be digested to monosaccharides prior to absorption in the small intestine (Kanehisa et al., 2017). Some fishes may able to digest mono‐, di‐, and oligosaccharides but not for indigestible complex carbohydrates such as hemicellulose and cellulose which usually plenty in plants (Krogdahl, Hemre, & Mommsen, 2005). High carbohydrate and high lipid diets have been widely used in aquaculture to reduce cost, but they also caused excessive lipid accumulation in the fish liver (Xie et al., 2017). In contrast, wild *T. tambroides* may consume algae, fruits, small fishes, and crustaceans. Thus, the gut microbiota in captive *T. tambroides* showed higher carbohydrate metabolism function. Many *Bacteroides* spp. such as *Bacteroides thetaiotaomicron* are capable of metabolize polysaccharides in gut (Ravcheev, Godzik, Osterman, & Rodionov, 2013). Besides, these bacteria also can provide energy from indigestible polysaccharides comprising part of the host diet (Schwalm & Groisman, 2017).

Lysine biosynthesis evolved separately into two pathways which are diaminopimelic acid (DAP) and aminoadipic acid (AAA) pathways (Liu, White, & Whitman, 2010). Lysine is an essential amino acid for living organism especially those consume vegetarian or low animal protein diet. Lysine biosynthesis functions were higher in wild *T. tambroides* gut microbiota, and this could be due to consumption of microalgae that was abundant in Kenyir Lake. PICRUSt predictions were made based on available genome sequences of bacteria thus some OTUs that lack of closely related genomes may be underpredicted (Salinas & Magadán, 2017). Lysine produced by gut microbiota was reported to be absorbed at the host's small intestines (Metges, 2000). *Bacillus sphaericus* and *Bacillus megaterium* were reported to have the capability to synthesize lysine (Bartlett & White, 1986; Ekwealor & Obeta, 2005).

In conclusion, species diversity was higher in wild *T. tambroides* gut microbiota as compared to captive *T. tambroides*. The samples of wild and captive *T. tambroides* gut microbiota could be clustered in the PCoA plots based on origin of the samples where the gut microbial composition of wild *T. tambroides* was different compared to captive *T. tambroides*. The results suggested that *Cetobacterium* spp. is one of the core microbiota in guts of *T. tambroides*. The other bacteria may be important in *T. tambroides* guts included those presented in high abundance, like *Bacteroides* spp., *Citrobacter* spp., *Turicibacter* spp., and *Bacillus* spp. Metagenetic sequencing in this study revealed much bacteria existed in the guts of wild and captive *T. tambroides*, and future research could be focused on isolate those bacteria that may be use as potential probiotics. This requires the development of specific media and analysis of growing conditions.

## CONFLICT OF INTEREST

The authors declare that they have no conflict of interests.

## AUTHORS CONTRIBUTION

Anjas and Tan conceived of the study conception and experimental design. Tan, Marilyn, and Nazrien were responsible for the acquisition of data. Anjas, Tan, Natrah, and Iswan analyzed and interpreted the data. Anjas and Tan drafted the manuscript. All authors discussed the results and contributed to the final manuscript.

## ETHICAL STATEMENT

This study was approved by Malaysia Institute Pharmaceuticals and Nutraceuticals Animal Ethic Committee (IPAEC) with approval number of NIBM/IPharm/PTR (S) 100‐7/7‐ 4 (2017‐004).

## Data Availability

All sequences were also submitted to NCBI Sequence Read Archive (SRA) under accession number of https://www.ncbi.nlm.nih.gov/bioproject/PRJNA355218/.
