# Supplementary material for: Comparative study of gut microbiota in wild and captive Malaysian Mahseer (Tor tambroides)
Source: Microbiologyopen. 2018 Oct 23;8(5):e00734. doi: 10.1002/mbo3.734 (PMC6528585; doi:10.1002/mbo3.734)
Supplement: Supplementary file 1 [file MBO3-8-e00734-s001.docx]

**SUPPLEMENTARY TABLE A: Complete list of OTUs and their percentages in wild and captive samples**

| # Constructed from biom file | | (%) | (%) |
| --- | --- | --- | --- |
| #OTU ID | | Wild | Captive |
| k__Bacteria;p__Fusobacteria;c__Fusobacteriia;o__Fusobacteriales;f__Fusobacteriaceae;g__Cetobacterium | 23.48 | | 29.07 |
| k__Bacteria;p__Firmicutes;c__Clostridia;o__Clostridiales;f__Peptostreptococcaceae;g__ | 11.87 | | 7.66 |
| k__Bacteria;p__Bacteroidetes;c__Bacteroidia;o__Bacteroidales;f__Bacteroidaceae;g__Bacteroides | 9.60 | | 1.78 |
| k__Bacteria;p__Firmicutes;c__Erysipelotrichi;o__Erysipelotrichales;f__Erysipelotrichaceae;g__PSB-M-3 | 7.70 | | 0.08 |
| k__Bacteria;p__Proteobacteria;c__Gammaproteobacteria;o__Vibrionales;f__Vibrionaceae;g__Vibrio | 4.94 | | 4.72 |
| k__Bacteria;p__Bacteroidetes;c__Bacteroidia;o__Bacteroidales;f__Bacteroidaceae;g__ | 3.89 | | 3.34 |
| k__Bacteria;p__Chloroflexi;c__Anaerolineae;o__Caldilineales;f__Caldilineaceae;g__Caldilinea | 3.50 | | 0.01 |
| k__Bacteria;p__Tenericutes;c__CK-1C4-19;o__;f__;g__ | 3.14 | | 0.81 |
| k__Bacteria;p__Proteobacteria;c__Alphaproteobacteria;o__Rhizobiales;f__;g__ | 2.91 | | 0.44 |
| k__Bacteria;p__Proteobacteria;c__Gammaproteobacteria;o__Aeromonadales;f__Aeromonadaceae;g__ | | 2.42 | 8.63 |
| k__Bacteria;p__Proteobacteria;c__Gammaproteobacteria;o__Enterobacteriales;f__Enterobacteriaceae;Other | | 2.27 | 1.18 |
| k__Bacteria;p__Firmicutes;c__Clostridia;o__Clostridiales;f__Clostridiaceae;g__Clostridium | | 2.09 | 0.42 |
| k__Bacteria;p__Proteobacteria;c__Alphaproteobacteria;o__Rhizobiales;f__Bradyrhizobiaceae;g__ | | 1.82 | 0.24 |
| k__Bacteria;p__Firmicutes;c__Clostridia;o__Clostridiales;f__Clostridiaceae;g__ | | 1.54 | 1.63 |
| k__Bacteria;p__Firmicutes;c__Clostridia;o__Clostridiales;f__Lachnospiraceae;g__Epulopiscium | | 1.23 | 3.78 |
| k__Bacteria;p__Proteobacteria;c__Alphaproteobacteria;o__Rhodobacterales;f__Rhodobacteraceae;g__Rhodobacter | | 1.14 | 0.01 |
| k__Bacteria;p__Actinobacteria;c__Acidimicrobiia;o__Acidimicrobiales;f__C111;g__ | | 1.13 | 0.00 |
| k__Bacteria;p__Actinobacteria;c__Actinobacteria;o__Actinomycetales;f__;g__ | | 1.04 | 0.25 |
| k__Bacteria;p__Bacteroidetes;c__Bacteroidia;o__Bacteroidales;f__Porphyromonadaceae;g__Paludibacter | | 0.89 | 1.39 |
| k__Bacteria;p__Planctomycetes;c__Planctomycetia;o__Gemmatales;f__Gemmataceae;g__ | | 0.76 | 0.01 |
| k__Bacteria;p__Firmicutes;c__Bacilli;o__Turicibacterales;f__Turicibacteraceae;g__Turicibacter | | 0.63 | 6.47 |
| k__Bacteria;p__Fusobacteria;c__Fusobacteriia;o__Fusobacteriales;f__Fusobacteriaceae;Other | | 0.61 | 0.07 |
| k__Bacteria;p__Proteobacteria;c__Alphaproteobacteria;o__Rhodospirillales;f__Rhodospirillaceae;g__ | | 0.55 | 2.05 |
| k__Bacteria;p__Planctomycetes;c__Planctomycetia;o__Pirellulales;f__Pirellulaceae;g__ | | 0.53 | 0.23 |
| k__Bacteria;p__Cyanobacteria;c__Chloroplast;o__Stramenopiles;f__;g__ | | 0.38 | 0.00 |
| k__Bacteria;p__Planctomycetes;c__Planctomycetia;o__Gemmatales;f__Isosphaeraceae;g__ | | 0.38 | 0.23 |
| k__Bacteria;p__Actinobacteria;c__Actinobacteria;o__Actinomycetales;Other;Other | | 0.37 | 0.01 |
| Unassigned;Other;Other;Other;Other;Other | | 0.37 | 0.10 |
| k__Bacteria;p__TM7;c__TM7-1;o__;f__;g__ | | 0.34 | 0.22 |
| k__Bacteria;p__Cyanobacteria;c__Synechococcophycideae;o__Pseudanabaenales;f__Pseudanabaenaceae;g__ | | 0.32 | 0.00 |
| k__Bacteria;p__Proteobacteria;c__Gammaproteobacteria;o__Enterobacteriales;f__Enterobacteriaceae;g__ | | 0.31 | 0.32 |
| k__Bacteria;p__Cyanobacteria;c__Synechococcophycideae;o__Synechococcales;f__Synechococcaceae;g__Synechococcus | | 0.31 | 0.00 |
| k__Bacteria;p__Proteobacteria;c__Alphaproteobacteria;o__Rhizobiales;f__Methylocystaceae;g__ | | 0.31 | 0.03 |
| k__Bacteria;p__Firmicutes;c__Bacilli;o__Lactobacillales;f__Enterococcaceae;g__Enterococcus | | 0.30 | 0.00 |
| k__Bacteria;p__Chloroflexi;c__Anaerolineae;o__SJA-15;f__;g__ | | 0.27 | 0.00 |
| k__Bacteria;p__Firmicutes;c__Clostridia;o__Clostridiales;f__Ruminococcaceae;g__ | | 0.24 | 0.04 |
| k__Bacteria;p__Actinobacteria;c__OPB41;o__;f__;g__ | | 0.24 | 0.00 |
| k__Bacteria;p__Planctomycetes;c__Planctomycetia;o__Planctomycetales;f__Planctomycetaceae;g__Planctomyces | | 0.20 | 0.23 |
| k__Bacteria;p__Cyanobacteria;c__Chloroplast;o__Streptophyta;f__;g__ | | 0.20 | 1.17 |
| k__Bacteria;p__Proteobacteria;c__Betaproteobacteria;o__SC-I-84;f__;g__ | | 0.20 | 0.00 |
| k__Bacteria;p__Chloroflexi;c__Anaerolineae;o__SHA-20;f__;g__ | | 0.18 | 0.00 |
| k__Bacteria;p__Firmicutes;c__Clostridia;o__Clostridiales;f__;g__ | | 0.18 | 0.04 |
| k__Bacteria;p__Actinobacteria;c__Acidimicrobiia;o__Acidimicrobiales;f__EB1017;g__ | | 0.18 | 0.04 |
| k__Bacteria;p__Actinobacteria;c__Acidimicrobiia;o__Acidimicrobiales;f__;g__ | | 0.18 | 0.11 |
| k__Bacteria;p__Firmicutes;c__Clostridia;o__Clostridiales;f__Peptostreptococcaceae;g__Peptostreptococcus | | 0.16 | 0.05 |
| k__Bacteria;p__Firmicutes;c__Clostridia;o__Clostridiales;f__Peptostreptococcaceae;Other | | 0.15 | 0.20 |
| k__Bacteria;p__Proteobacteria;c__Alphaproteobacteria;o__Rhodospirillales;f__Acetobacteraceae;g__ | | 0.15 | 0.10 |
| k__Bacteria;p__Planctomycetes;c__Planctomycetia;o__Gemmatales;f__Gemmataceae;g__Gemmata | | 0.14 | 0.09 |
| k__Bacteria;p__Firmicutes;c__Bacilli;o__Lactobacillales;f__Streptococcaceae;g__Streptococcus | | 0.14 | 0.02 |
| k__Bacteria;p__Chloroflexi;c__Anaerolineae;o__;f__;g__ | | 0.13 | 0.00 |
| k__Bacteria;p__Firmicutes;c__Clostridia;o__Clostridiales;f__Lachnospiraceae;g__ | | 0.12 | 0.01 |
| k__Bacteria;p__Firmicutes;c__Erysipelotrichi;o__Erysipelotrichales;f__Erysipelotrichaceae;Other | | 0.12 | 0.00 |
| k__Bacteria;p__Proteobacteria;c__Betaproteobacteria;Other;Other;Other | | 0.11 | 0.00 |
| k__Bacteria;p__Proteobacteria;c__Gammaproteobacteria;o__Methylococcales;f__Methylococcaceae;g__Methylocaldum | | 0.11 | 0.00 |
| k__Bacteria;p__Planctomycetes;c__Planctomycetia;o__Pirellulales;f__Pirellulaceae;g__Pirellula | | 0.10 | 0.01 |
| k__Bacteria;p__Verrucomicrobia;c__Verrucomicrobiae;o__Verrucomicrobiales;f__Verrucomicrobiaceae;g__ | | 0.10 | 0.00 |
| k__Bacteria;p__Proteobacteria;c__Gammaproteobacteria;o__HOC36;f__;g__ | | 0.10 | 0.00 |
| k__Bacteria;p__Actinobacteria;c__Actinobacteria;o__Actinomycetales;f__Mycobacteriaceae;g__Mycobacterium | | 0.10 | 0.16 |
| k__Bacteria;p__Proteobacteria;c__Alphaproteobacteria;o__Rhizobiales;f__Methylocystaceae;g__Methylosinus | | 0.09 | 0.00 |
| k__Bacteria;p__Proteobacteria;c__Alphaproteobacteria;o__Rhizobiales;f__Hyphomicrobiaceae;g__Rhodoplanes | | 0.09 | 0.08 |
| k__Bacteria;p__Proteobacteria;c__Betaproteobacteria;o__;f__;g__ | | 0.08 | 0.00 |
| k__Bacteria;p__Actinobacteria;c__Actinobacteria;o__Actinomycetales;f__Actinomycetaceae;g__Actinomyces | | 0.08 | 0.06 |
| k__Bacteria;p__Acidobacteria;c__Acidobacteria-6;o__CCU21;f__;g__ | | 0.08 | 0.00 |
| k__Bacteria;p__Verrucomicrobia;c__Verrucomicrobiae;o__Verrucomicrobiales;f__Verrucomicrobiaceae;g__Akkermansia | | 0.08 | 0.02 |
| k__Bacteria;p__Proteobacteria;c__Betaproteobacteria;o__ASSO-13;f__;g__ | | 0.08 | 0.00 |
| k__Bacteria;p__Bacteroidetes;c__Bacteroidia;o__Bacteroidales;f__Bacteroidaceae;Other | | 0.08 | 0.01 |
| k__Bacteria;p__Chloroflexi;c__Anaerolineae;o__SBR1031;f__A4b;g__ | | 0.07 | 0.00 |
| k__Bacteria;p__Proteobacteria;c__Deltaproteobacteria;o__Syntrophobacterales;f__Syntrophobacteraceae;g__ | | 0.07 | 0.01 |
| k__Bacteria;p__Proteobacteria;c__Alphaproteobacteria;o__Rhizobiales;f__Methylocystaceae;Other | | 0.06 | 0.00 |
| k__Bacteria;p__Chloroflexi;c__Anaerolineae;o__WCHB1-50;f__;g__ | | 0.06 | 0.00 |
| k__Bacteria;p__Firmicutes;c__Clostridia;o__Clostridiales;f__Lachnospiraceae;g__Coprococcus | | 0.05 | 0.00 |
| k__Bacteria;p__Verrucomicrobia;c__[Spartobacteria];o__[Chthoniobacterales];f__[Chthoniobacteraceae];g__Candidatus Xiphinematobacter | | 0.05 | 0.13 |
| k__Bacteria;p__Firmicutes;c__Clostridia;o__Clostridiales;f__Ruminococcaceae;g__Oscillospira | | 0.05 | 0.01 |
| k__Bacteria;p__Firmicutes;c__Clostridia;o__Clostridiales;f__Ruminococcaceae;g__Faecalibacterium | | 0.05 | 0.00 |
| k__Bacteria;p__Actinobacteria;c__Actinobacteria;o__Actinomycetales;f__Microbacteriaceae;g__Leucobacter | | 0.05 | 0.23 |
| k__Bacteria;p__Cyanobacteria;c__Synechococcophycideae;o__Pseudanabaenales;f__Pseudanabaenaceae;Other | | 0.05 | 0.00 |
| k__Bacteria;p__Firmicutes;c__Clostridia;o__Clostridiales;f__Clostridiaceae;g__Proteiniclasticum | | 0.04 | 0.00 |
| k__Bacteria;p__Proteobacteria;c__Alphaproteobacteria;o__Rhizobiales;f__Bradyrhizobiaceae;g__Balneimonas | | 0.04 | 0.00 |
| k__Bacteria;p__Firmicutes;c__Clostridia;o__Clostridiales;f__Peptococcaceae;g__ | | 0.04 | 0.00 |
| k__Bacteria;p__Actinobacteria;c__Actinobacteria;o__Actinomycetales;f__Microbacteriaceae;Other | | 0.04 | 1.74 |
| k__Bacteria;p__Proteobacteria;c__Deltaproteobacteria;o__Desulfovibrionales;f__Desulfovibrionaceae;g__ | | 0.04 | 0.00 |
| k__Bacteria;p__Cyanobacteria;c__Synechococcophycideae;o__Pseudanabaenales;Other;Other | | 0.04 | 0.00 |
| k__Bacteria;p__Chloroflexi;c__Thermomicrobia;o__JG30-KF-CM45;f__;g__ | | 0.04 | 0.26 |
| k__Bacteria;p__Firmicutes;c__Bacilli;o__Bacillales;f__Bacillaceae;g__Bacillus | | 0.04 | 2.52 |
| k__Bacteria;p__Firmicutes;c__Erysipelotrichi;o__Erysipelotrichales;f__Erysipelotrichaceae;g__ | | 0.04 | 0.00 |
| k__Bacteria;p__Proteobacteria;c__Alphaproteobacteria;o__Rhizobiales;f__Hyphomicrobiaceae;g__Hyphomicrobium | | 0.04 | 0.06 |
| k__Bacteria;p__Proteobacteria;c__Gammaproteobacteria;o__Legionellales;f__Legionellaceae;g__ | | 0.04 | 0.18 |
| k__Bacteria;p__Firmicutes;c__Clostridia;o__Clostridiales;Other;Other | | 0.04 | 0.04 |
| k__Bacteria;p__Proteobacteria;c__Betaproteobacteria;o__Rhodocyclales;f__Rhodocyclaceae;g__Dechloromonas | | 0.03 | 0.00 |
| k__Bacteria;p__Proteobacteria;c__Gammaproteobacteria;o__Alteromonadales;f__Shewanellaceae;g__Shewanella | | 0.03 | 0.13 |
| k__Bacteria;p__Proteobacteria;c__Alphaproteobacteria;o__Rhodobacterales;f__Rhodobacteraceae;g__ | | 0.03 | 0.06 |
| k__Bacteria;p__Proteobacteria;c__Gammaproteobacteria;o__Legionellales;f__Coxiellaceae;g__ | | 0.03 | 0.08 |
| k__Bacteria;p__Planctomycetes;c__Planctomycetia;o__Pirellulales;f__Pirellulaceae;g__A17 | | 0.03 | 0.05 |
| k__Bacteria;p__Actinobacteria;c__Acidimicrobiia;o__Acidimicrobiales;f__Microthrixaceae;g__ | | 0.03 | 0.00 |
| k__Bacteria;p__Firmicutes;c__Clostridia;o__Clostridiales;f__Clostridiaceae;Other | | 0.03 | 0.06 |
| k__Bacteria;p__Proteobacteria;c__Alphaproteobacteria;o__Rhizobiales;f__Bradyrhizobiaceae;Other | | 0.03 | 0.01 |
| k__Bacteria;p__Chloroflexi;c__Anaerolineae;o__A31;f__S47;g__ | | 0.03 | 0.00 |
| k__Bacteria;p__Chloroflexi;c__Chloroflexi;o__Chloroflexales;f__Chloroflexaceae;Other | | 0.03 | 0.00 |
| k__Bacteria;p__Chloroflexi;c__Anaerolineae;o__GCA004;f__;g__ | | 0.03 | 0.00 |
| k__Bacteria;p__Proteobacteria;c__Gammaproteobacteria;o__Vibrionales;f__Pseudoalteromonadaceae;Other | | 0.03 | 0.03 |
| k__Bacteria;p__Chloroflexi;c__Anaerolineae;o__Caldilineales;f__Caldilineaceae;g__ | | 0.03 | 0.00 |
| k__Bacteria;p__TM7;c__SC3;o__;f__;g__ | | 0.03 | 0.02 |
| k__Bacteria;p__Firmicutes;c__Bacilli;o__Lactobacillales;f__Streptococcaceae;g__Lactococcus | | 0.02 | 0.00 |
| k__Bacteria;p__Firmicutes;c__Clostridia;o__Clostridiales;f__Clostridiaceae;g__SMB53 | | 0.02 | 0.03 |
| k__Bacteria;p__Proteobacteria;c__Gammaproteobacteria;o__Vibrionales;f__Pseudoalteromonadaceae;g__ | | 0.02 | 0.03 |
| k__Bacteria;p__Firmicutes;c__Clostridia;o__Clostridiales;f__Ruminococcaceae;g__Ruminococcus | | 0.02 | 0.01 |
| k__Bacteria;p__Proteobacteria;c__Alphaproteobacteria;o__Rhodospirillales;f__;g__ | | 0.02 | 0.02 |
| k__Bacteria;p__Proteobacteria;c__Alphaproteobacteria;o__Rhizobiales;f__Methylobacteriaceae;g__Methylobacterium | | 0.02 | 0.00 |
| k__Bacteria;p__Proteobacteria;c__Alphaproteobacteria;o__Rhizobiales;f__Rhizobiaceae;g__Agrobacterium | | 0.02 | 0.03 |
| k__Bacteria;p__Proteobacteria;c__Gammaproteobacteria;o__Enterobacteriales;f__Enterobacteriaceae;g__Plesiomonas | | 0.02 | 0.02 |
| k__Bacteria;p__Proteobacteria;c__Gammaproteobacteria;o__Enterobacteriales;f__Enterobacteriaceae;g__Citrobacter | | 0.02 | 9.35 |
| k__Bacteria;p__Actinobacteria;c__Actinobacteria;o__Actinomycetales;f__Pseudonocardiaceae;Other | | 0.02 | 0.00 |
| k__Bacteria;p__Proteobacteria;c__Alphaproteobacteria;o__Rhizobiales;f__Beijerinckiaceae;g__ | | 0.02 | 0.00 |
| k__Bacteria;p__Actinobacteria;c__Thermoleophilia;o__Solirubrobacterales;f__Patulibacteraceae;g__ | | 0.02 | 0.00 |
| k__Bacteria;p__Cyanobacteria;c__Chloroplast;o__Chlorophyta;f__Chlamydomonadaceae;g__ | | 0.02 | 0.00 |
| k__Bacteria;p__Chloroflexi;c__Chloroflexi;o__Chloroflexales;f__Chloroflexaceae;g__Chloronema | | 0.02 | 0.00 |
| k__Bacteria;p__Proteobacteria;c__Alphaproteobacteria;o__Rhizobiales;f__Bradyrhizobiaceae;g__Bosea | | 0.02 | 0.03 |
| k__Bacteria;p__Proteobacteria;c__Betaproteobacteria;o__Burkholderiales;f__Alcaligenaceae;g__ | | 0.02 | 0.00 |
| k__Bacteria;p__Bacteroidetes;c__[Saprospirae];o__[Saprospirales];f__Chitinophagaceae;g__ | | 0.02 | 0.00 |
| k__Bacteria;p__Proteobacteria;c__Gammaproteobacteria;o__Vibrionales;Other;Other | | 0.02 | 0.03 |
| k__Bacteria;p__Actinobacteria;c__Actinobacteria;o__Actinomycetales;f__Microbacteriaceae;g__Cryocola | | 0.02 | 0.92 |
| k__Bacteria;p__Proteobacteria;c__Alphaproteobacteria;o__Rhizobiales;f__Hyphomicrobiaceae;g__ | | 0.01 | 0.01 |
| k__Bacteria;p__Proteobacteria;c__Deltaproteobacteria;o__Bdellovibrionales;f__Bdellovibrionaceae;g__Bdellovibrio | | 0.01 | 0.00 |
| k__Bacteria;p__Chloroflexi;c__Ellin6529;o__;f__;g__ | | 0.01 | 0.00 |
| k__Bacteria;p__Proteobacteria;c__Gammaproteobacteria;o__Methylococcales;f__Methylococcaceae;Other | | 0.01 | 0.00 |
| k__Bacteria;p__Proteobacteria;c__Gammaproteobacteria;o__Vibrionales;f__Vibrionaceae;Other | | 0.01 | 0.01 |
| k__Bacteria;p__Cyanobacteria;c__Synechococcophycideae;o__Pseudanabaenales;f__Pseudanabaenaceae;g__Arthronema | | 0.01 | 0.00 |
| k__Bacteria;p__Proteobacteria;c__Alphaproteobacteria;o__Rhodobacterales;f__Rhodobacteraceae;g__Anaerospora | | 0.01 | 0.00 |
| k__Bacteria;p__Actinobacteria;c__Actinobacteria;o__Actinomycetales;f__Microbacteriaceae;g__ | | 0.01 | 0.02 |
| k__Bacteria;p__Lentisphaerae;c__[Lentisphaeria];o__Lentisphaerales;f__;g__ | | 0.01 | 0.00 |
| k__Bacteria;p__TM7;c__TM7-3;o__;f__;g__ | | 0.01 | 0.03 |
| k__Bacteria;p__Verrucomicrobia;c__[Spartobacteria];o__[Chthoniobacterales];f__[Chthoniobacteraceae];g__DA101 | | 0.01 | 0.00 |
| k__Bacteria;p__Verrucomicrobia;c__Verrucomicrobiae;o__Verrucomicrobiales;f__Verrucomicrobiaceae;g__Luteolibacter | | 0.01 | 0.01 |
| k__Bacteria;p__Planctomycetes;c__Planctomycetia;o__B97;f__;g__ | | 0.01 | 0.02 |
| k__Bacteria;p__Proteobacteria;c__Gammaproteobacteria;o__Xanthomonadales;f__Sinobacteraceae;g__ | | 0.01 | 0.00 |
| k__Bacteria;p__Proteobacteria;c__Alphaproteobacteria;o__Rhizobiales;f__Brucellaceae;g__Ochrobactrum | | 0.01 | 0.40 |
| k__Bacteria;p__Cyanobacteria;c__Synechococcophycideae;o__Pseudanabaenales;f__Pseudanabaenaceae;g__Leptolyngbya | | 0.01 | 0.00 |
| k__Bacteria;p__Verrucomicrobia;c__[Spartobacteria];o__[Chthoniobacterales];f__[Chthoniobacteraceae];g__ | | 0.01 | 0.00 |
| k__Bacteria;p__Cyanobacteria;c__Synechococcophycideae;o__Synechococcales;f__Acaryochloridaceae;g__ | | 0.01 | 0.00 |
| k__Bacteria;p__Proteobacteria;c__Deltaproteobacteria;o__Myxococcales;f__;g__ | | 0.01 | 0.03 |
| k__Bacteria;p__Firmicutes;c__Clostridia;o__Clostridiales;f__Lachnospiraceae;g__[Ruminococcus] | | 0.01 | 0.00 |
| k__Bacteria;p__Proteobacteria;c__Betaproteobacteria;o__Burkholderiales;f__Comamonadaceae;g__ | | 0.01 | 0.01 |
| k__Bacteria;p__Proteobacteria;c__Betaproteobacteria;o__Neisseriales;f__Neisseriaceae;g__ | | 0.01 | 0.00 |
| k__Bacteria;p__Proteobacteria;c__Gammaproteobacteria;o__Legionellales;f__Coxiellaceae;g__Aquicella | | 0.01 | 0.08 |
| k__Bacteria;p__Cyanobacteria;c__Chloroplast;o__Chlorophyta;f__;g__ | | 0.01 | 0.00 |
| k__Bacteria;p__Proteobacteria;c__Alphaproteobacteria;o__Rhizobiales;f__Aurantimonadaceae;g__ | | 0.01 | 0.00 |
| k__Bacteria;p__TM6;c__SJA-4;o__;f__;g__ | | 0.01 | 0.26 |
| k__Bacteria;p__Proteobacteria;c__Alphaproteobacteria;o__Rhizobiales;Other;Other | | 0.01 | 0.00 |
| k__Bacteria;p__Proteobacteria;c__Gammaproteobacteria;o__Legionellales;f__Legionellaceae;g__Legionella | | 0.01 | 0.03 |
| k__Bacteria;p__Firmicutes;c__Clostridia;o__Clostridiales;f__Lachnospiraceae;Other | | 0.01 | 0.00 |
| k__Bacteria;p__Proteobacteria;c__Alphaproteobacteria;o__Rickettsiales;f__;g__ | | 0.01 | 0.18 |
| k__Bacteria;p__Firmicutes;c__Clostridia;o__Clostridiales;f__Peptococcaceae;Other | | 0.01 | 0.00 |
| k__Bacteria;p__Firmicutes;c__Bacilli;o__Bacillales;f__Bacillaceae;g__ | | 0.01 | 0.49 |
| k__Bacteria;p__Firmicutes;c__Clostridia;o__Clostridiales;f__Peptostreptococcaceae;g__Clostridium | | 0.01 | 0.01 |
| k__Bacteria;p__Proteobacteria;c__Alphaproteobacteria;o__Rhizobiales;f__Phyllobacteriaceae;g__ | | 0.01 | 0.00 |
| k__Bacteria;p__Proteobacteria;c__Gammaproteobacteria;o__Legionellales;f__;g__ | | 0.01 | 0.37 |
| k__Bacteria;p__Actinobacteria;c__Actinobacteria;o__Actinomycetales;f__Microbacteriaceae;g__Salinibacterium | | 0.01 | 0.37 |
| k__Bacteria;p__Synergistetes;c__Synergistia;o__Synergistales;f__Synergistaceae;Other | | 0.01 | 0.00 |
| k__Bacteria;p__Proteobacteria;c__Alphaproteobacteria;o__Rhodospirillales;f__Acetobacteraceae;g__Roseococcus | | 0.01 | 0.00 |
| k__Bacteria;p__Proteobacteria;c__Alphaproteobacteria;o__Rhizobiales;f__Methylobacteriaceae;g__ | | 0.01 | 0.00 |
| k__Bacteria;p__Actinobacteria;c__Thermoleophilia;o__Gaiellales;f__Gaiellaceae;g__ | | 0.01 | 0.00 |
| k__Bacteria;p__Acidobacteria;c__Acidobacteria-6;o__iii1-15;f__;g__ | | 0.01 | 0.00 |
| k__Bacteria;p__Actinobacteria;c__MB-A2-108;o__0319-7L14;f__;g__ | | 0.01 | 0.00 |
| k__Bacteria;p__Cyanobacteria;c__4C0d-2;o__YS2;f__;g__ | | 0.01 | 0.00 |
| k__Bacteria;p__Proteobacteria;c__Alphaproteobacteria;o__Rhizobiales;f__Methylobacteriaceae;Other | | 0.01 | 0.00 |
| k__Bacteria;p__Proteobacteria;c__Alphaproteobacteria;o__Rhizobiales;f__Xanthobacteraceae;g__Labrys | | 0.01 | 0.00 |
| k__Bacteria;p__Cyanobacteria;c__Oscillatoriophycideae;o__Chroococcales;f__;g__ | | 0.01 | 0.00 |
| k__Bacteria;p__Proteobacteria;c__Betaproteobacteria;o__Neisseriales;f__Neisseriaceae;g__Chitinilyticum | | 0.01 | 0.00 |
| k__Bacteria;p__Actinobacteria;c__Actinobacteria;o__Actinomycetales;f__Microbacteriaceae;g__Pseudoclavibacter | | 0.01 | 0.00 |
| k__Bacteria;p__Proteobacteria;c__Alphaproteobacteria;o__Rhizobiales;f__Phyllobacteriaceae;g__Mesorhizobium | | 0.01 | 0.01 |
| k__Bacteria;p__Firmicutes;c__Clostridia;o__Clostridiales;f__Lachnospiraceae;g__Blautia | | 0.01 | 0.00 |
| k__Bacteria;p__Proteobacteria;c__Betaproteobacteria;o__Neisseriales;f__Neisseriaceae;Other | | 0.01 | 0.00 |
| k__Bacteria;p__Bacteroidetes;c__Bacteroidia;o__Bacteroidales;f__Porphyromonadaceae;g__Dysgonomonas | | 0.00 | 0.00 |
| k__Bacteria;p__Actinobacteria;c__Thermoleophilia;o__Gaiellales;f__AK1AB1_02E;g__ | | 0.00 | 0.00 |
| k__Bacteria;p__WS5;c__;o__;f__;g__ | | 0.00 | 0.00 |
| k__Bacteria;p__Chloroflexi;c__Chloroflexi;o__Chloroflexales;f__Oscillochloridaceae;g__Oscillochloris | | 0.00 | 0.00 |
| k__Bacteria;p__Proteobacteria;c__Alphaproteobacteria;o__Rhizobiales;f__Methylocystaceae;g__Pleomorphomonas | | 0.00 | 0.00 |
| k__Bacteria;p__Bacteroidetes;c__Bacteroidia;o__Bacteroidales;f__[Odoribacteraceae];g__Odoribacter | | 0.00 | 0.00 |
| k__Bacteria;p__Proteobacteria;c__Alphaproteobacteria;o__Rhizobiales;f__Hyphomicrobiaceae;g__Devosia | | 0.00 | 0.01 |
| k__Bacteria;p__Proteobacteria;c__Betaproteobacteria;o__Tremblayales;f__;g__ | | 0.00 | 0.00 |
| k__Bacteria;p__Verrucomicrobia;c__[Pedosphaerae];o__[Pedosphaerales];f__;g__ | | 0.00 | 0.00 |
| k__Bacteria;p__Proteobacteria;c__Alphaproteobacteria;o__Sphingomonadales;f__Sphingomonadaceae;g__ | | 0.00 | 0.01 |
| k__Bacteria;p__Proteobacteria;c__Alphaproteobacteria;o__Rhizobiales;f__Xanthobacteraceae;Other | | 0.00 | 0.01 |
| k__Bacteria;p__Proteobacteria;c__Betaproteobacteria;o__Rhodocyclales;f__Rhodocyclaceae;Other | | 0.00 | 0.00 |
| k__Bacteria;p__Actinobacteria;c__Actinobacteria;o__Actinomycetales;f__Nocardioidaceae;g__ | | 0.00 | 0.01 |
| k__Bacteria;p__Proteobacteria;c__Alphaproteobacteria;o__Rhizobiales;f__Beijerinckiaceae;Other | | 0.00 | 0.00 |
| k__Bacteria;p__Cyanobacteria;c__4C0d-2;o__MLE1-12;f__;g__ | | 0.00 | 0.00 |
| k__Bacteria;p__Proteobacteria;c__Betaproteobacteria;o__Thiobacterales;f__;g__ | | 0.00 | 0.00 |
| k__Bacteria;p__Proteobacteria;c__Gammaproteobacteria;o__Alteromonadales;f__OM60;g__ | | 0.00 | 0.00 |
| k__Bacteria;p__Proteobacteria;c__Alphaproteobacteria;o__Sphingomonadales;f__Sphingomonadaceae;g__Kaistobacter | | 0.00 | 0.00 |
| k__Bacteria;p__Proteobacteria;c__Deltaproteobacteria;o__Spirobacillales;f__;g__ | | 0.00 | 0.00 |
| k__Bacteria;p__Nitrospirae;c__Nitrospira;o__Nitrospirales;f__0319-6A21;g__ | | 0.00 | 0.00 |
| k__Bacteria;p__Firmicutes;c__Bacilli;o__Lactobacillales;f__Enterococcaceae;Other | | 0.00 | 0.00 |
| k__Bacteria;p__Firmicutes;c__Bacilli;o__Bacillales;f__;g__ | | 0.00 | 0.00 |
| k__Bacteria;p__Bacteroidetes;c__Bacteroidia;o__Bacteroidales;f__Porphyromonadaceae;Other | | 0.00 | 0.00 |
| k__Bacteria;p__Chloroflexi;c__Anaerolineae;o__Caldilineales;f__Caldilineaceae;Other | | 0.00 | 0.00 |
| k__Bacteria;p__Firmicutes;c__Bacilli;o__Lactobacillales;f__Lactobacillaceae;g__Lactobacillus | | 0.00 | 0.02 |
| k__Bacteria;p__Tenericutes;c__Mollicutes;o__Anaeroplasmatales;f__Anaeroplasmataceae;g__Anaeroplasma | | 0.00 | 0.00 |
| k__Bacteria;p__Proteobacteria;c__Betaproteobacteria;o__Rhodocyclales;f__Rhodocyclaceae;g__C39 | | 0.00 | 0.00 |
| k__Bacteria;p__Actinobacteria;c__Thermoleophilia;o__Solirubrobacterales;f__;g__ | | 0.00 | 0.01 |
| k__Bacteria;p__Proteobacteria;c__Gammaproteobacteria;o__Methylococcales;f__Crenotrichaceae;g__Crenothrix | | 0.00 | 0.00 |
| k__Bacteria;p__Acidobacteria;c__Solibacteres;o__Solibacterales;f__Solibacteraceae;g__Candidatus Solibacter | | 0.00 | 0.00 |
| k__Bacteria;p__Proteobacteria;c__Alphaproteobacteria;o__Rhizobiales;f__Phyllobacteriaceae;g__Aminobacter | | 0.00 | 0.00 |
| k__Bacteria;p__Chloroflexi;c__Anaerolineae;o__Anaerolineales;f__Anaerolinaceae;g__ | | 0.00 | 0.00 |
| k__Bacteria;p__Proteobacteria;c__Betaproteobacteria;o__Burkholderiales;f__Alcaligenaceae;g__Achromobacter | | 0.00 | 0.00 |
| k__Bacteria;p__Actinobacteria;c__Actinobacteria;o__Actinomycetales;f__Intrasporangiaceae;g__Arsenicicoccus | | 0.00 | 0.00 |
| k__Bacteria;p__Actinobacteria;c__Actinobacteria;o__Actinomycetales;f__Propionibacteriaceae;g__ | | 0.00 | 0.00 |
| k__Archaea;p__Euryarchaeota;c__Methanobacteria;o__Methanobacteriales;f__Methanobacteriaceae;g__Methanobacterium | | 0.00 | 0.00 |
| k__Bacteria;p__Proteobacteria;c__Alphaproteobacteria;o__Rickettsiales;f__mitochondria;Other | | 0.00 | 0.25 |
| k__Bacteria;p__Firmicutes;c__Bacilli;o__Bacillales;f__Thermoactinomycetaceae;g__ | | 0.00 | 0.00 |
| k__Bacteria;p__Proteobacteria;c__Deltaproteobacteria;o__FAC87;f__;g__ | | 0.00 | 0.00 |
| k__Bacteria;p__Proteobacteria;c__Alphaproteobacteria;o__Rhizobiales;f__Rhizobiaceae;g__ | | 0.00 | 0.15 |
| k__Bacteria;p__Proteobacteria;c__Alphaproteobacteria;o__Rhizobiales;f__Rhizobiaceae;g__Rhizobium | | 0.00 | 0.00 |
| k__Bacteria;p__Firmicutes;c__Clostridia;o__Clostridiales;f__Ruminococcaceae;g__Anaerotruncus | | 0.00 | 0.00 |
| k__Bacteria;p__Actinobacteria;c__Actinobacteria;o__Actinomycetales;f__Nocardiaceae;g__Rhodococcus | | 0.00 | 0.00 |
| k__Bacteria;p__WPS-2;c__;o__;f__;g__ | | 0.00 | 0.00 |
| k__Bacteria;p__Proteobacteria;c__Gammaproteobacteria;o__Pseudomonadales;f__Pseudomonadaceae;g__Pseudomonas | | 0.00 | 0.01 |
| k__Bacteria;p__Proteobacteria;c__Deltaproteobacteria;o__;f__;g__ | | 0.00 | 0.00 |
| k__Bacteria;p__Firmicutes;c__Erysipelotrichi;o__Erysipelotrichales;f__Erysipelotrichaceae;g__Coprobacillus | | 0.00 | 0.00 |
| k__Bacteria;p__Chloroflexi;c__Anaerolineae;o__pLW-97;f__;g__ | | 0.00 | 0.00 |
| k__Bacteria;p__Verrucomicrobia;c__[Spartobacteria];o__[Chthoniobacterales];f__[Chthoniobacteraceae];g__Chthoniobacter | | 0.00 | 0.00 |
| k__Bacteria;p__Fusobacteria;c__Fusobacteriia;o__Fusobacteriales;f__Fusobacteriaceae;g__Fusobacterium | | 0.00 | 0.02 |
| k__Bacteria;p__Proteobacteria;c__Alphaproteobacteria;o__Rhodobacterales;f__Rhodobacteraceae;Other | | 0.00 | 0.00 |
| k__Bacteria;p__Proteobacteria;c__Gammaproteobacteria;o__Legionellales;f__Legionellaceae;g__Tatlockia | | 0.00 | 0.01 |
| k__Bacteria;p__Actinobacteria;c__Rubrobacteria;o__Rubrobacterales;f__Rubrobacteraceae;g__Rubrobacter | | 0.00 | 0.00 |
| k__Bacteria;p__Actinobacteria;c__Actinobacteria;o__Actinomycetales;f__Promicromonosporaceae;Other | | 0.00 | 0.00 |
| k__Bacteria;p__Proteobacteria;c__Gammaproteobacteria;o__Pseudomonadales;f__Moraxellaceae;g__Enhydrobacter | | 0.00 | 0.00 |
| k__Bacteria;p__Planctomycetes;c__Phycisphaerae;o__WD2101;f__;g__ | | 0.00 | 0.00 |
| k__Bacteria;p__Chlamydiae;c__Chlamydiia;o__Chlamydiales;f__Parachlamydiaceae;g__Parachlamydia | | 0.00 | 0.02 |
| k__Bacteria;p__Proteobacteria;c__Alphaproteobacteria;o__Rhizobiales;f__Xanthobacteraceae;g__ | | 0.00 | 0.00 |
| k__Bacteria;p__BRC1;c__PRR-11;o__;f__;g__ | | 0.00 | 0.00 |
| k__Bacteria;p__TM7;c__TM7-3;o__Blgi18;f__;g__ | | 0.00 | 0.00 |
| k__Bacteria;p__Proteobacteria;c__Gammaproteobacteria;o__Xanthomonadales;f__Xanthomonadaceae;g__ | | 0.00 | 1.31 |
| k__Bacteria;p__Acidobacteria;c__Solibacteres;o__Solibacterales;f__;g__ | | 0.00 | 0.00 |
| k__Bacteria;p__Proteobacteria;c__Alphaproteobacteria;o__Rhodospirillales;f__Acetobacteraceae;g__Roseomonas | | 0.00 | 0.00 |
| k__Bacteria;p__Synergistetes;c__Synergistia;o__Synergistales;f__Dethiosulfovibrionaceae;g__TG5 | | 0.00 | 0.00 |
| k__Bacteria;p__Actinobacteria;c__Actinobacteria;o__Actinomycetales;f__Microbacteriaceae;g__Microbacterium | | 0.00 | 0.02 |
| k__Bacteria;p__Firmicutes;c__Clostridia;o__Clostridiales;f__Lachnospiraceae;g__Dorea | | 0.00 | 0.00 |
| k__Bacteria;p__Proteobacteria;c__Gammaproteobacteria;o__Legionellales;f__Legionellaceae;Other | | 0.00 | 0.00 |
| k__Bacteria;p__Proteobacteria;c__Deltaproteobacteria;o__NB1-j;f__;g__ | | 0.00 | 0.00 |
| k__Bacteria;p__Proteobacteria;c__Alphaproteobacteria;o__Sphingomonadales;f__Sphingomonadaceae;g__Novosphingobium | | 0.00 | 0.02 |
| k__Bacteria;p__Proteobacteria;c__Gammaproteobacteria;o__Thiotrichales;f__Piscirickettsiaceae;g__ | | 0.00 | 0.00 |
| k__Bacteria;p__Proteobacteria;c__Alphaproteobacteria;o__Rhizobiales;f__Hyphomicrobiaceae;g__Pedomicrobium | | 0.00 | 0.00 |
| k__Bacteria;p__Cyanobacteria;c__Oscillatoriophycideae;Other;Other;Other | | 0.00 | 0.00 |
| k__Bacteria;p__TM7;c__TM7-3;o__EW055;f__;g__ | | 0.00 | 0.03 |
| k__Bacteria;p__Proteobacteria;c__Alphaproteobacteria;o__Rhizobiales;f__Phyllobacteriaceae;Other | | 0.00 | 0.00 |
| k__Bacteria;p__Actinobacteria;c__Actinobacteria;o__Actinomycetales;f__Nocardioidaceae;g__Aeromicrobium | | 0.00 | 0.00 |
| k__Bacteria;p__GAL15;c__;o__;f__;g__ | | 0.00 | 0.00 |
| k__Bacteria;p__Verrucomicrobia;c__[Pedosphaerae];o__[Pedosphaerales];f__Ellin515;g__ | | 0.00 | 0.00 |
| k__Bacteria;p__Firmicutes;c__Bacilli;o__Bacillales;f__Planococcaceae;g__Planomicrobium | | 0.00 | 0.00 |
| k__Bacteria;p__Proteobacteria;c__Betaproteobacteria;o__Neisseriales;f__Neisseriaceae;g__Deefgea | | 0.00 | 0.00 |
| k__Bacteria;p__Proteobacteria;c__Gammaproteobacteria;o__Legionellales;f__Coxiellaceae;g__Rickettsiella | | 0.00 | 0.00 |
| k__Bacteria;p__Proteobacteria;c__Deltaproteobacteria;o__Syntrophobacterales;f__Syntrophaceae;g__Desulfobacca | | 0.00 | 0.00 |
| k__Bacteria;p__Chlamydiae;c__Chlamydiia;o__Chlamydiales;f__;g__ | | 0.00 | 0.02 |
| k__Bacteria;p__Proteobacteria;c__Betaproteobacteria;o__Burkholderiales;f__Comamonadaceae;Other | | 0.00 | 0.01 |
| k__Bacteria;p__Proteobacteria;c__Alphaproteobacteria;o__Sphingomonadales;f__Sphingomonadaceae;Other | | 0.00 | 0.00 |
| k__Bacteria;p__Tenericutes;c__Mollicutes;o__Mycoplasmatales;f__Mycoplasmataceae;g__Mycoplasma | | 0.00 | 0.00 |
| k__Bacteria;p__Actinobacteria;c__Actinobacteria;o__Actinomycetales;f__Nocardioidaceae;g__Pimelobacter | | 0.00 | 0.00 |
| k__Bacteria;p__Proteobacteria;c__Betaproteobacteria;o__Rhodocyclales;f__Rhodocyclaceae;g__Dok59 | | 0.00 | 0.00 |
| k__Bacteria;p__Proteobacteria;c__TA18;o__PHOS-HD29;f__;g__ | | 0.00 | 0.00 |
| k__Bacteria;p__Proteobacteria;c__Alphaproteobacteria;o__;f__;g__ | | 0.00 | 0.00 |
| k__Bacteria;p__Proteobacteria;c__Alphaproteobacteria;o__Rhizobiales;f__Hyphomicrobiaceae;Other | | 0.00 | 0.00 |
| k__Bacteria;p__Firmicutes;c__Bacilli;o__Bacillales;f__Alicyclobacillaceae;g__Alicyclobacillus | | 0.00 | 0.00 |
| k__Bacteria;p__Actinobacteria;c__Actinobacteria;o__Actinomycetales;f__Nakamurellaceae;g__ | | 0.00 | 0.00 |
| k__Bacteria;p__Actinobacteria;c__Acidimicrobiia;o__Acidimicrobiales;Other;Other | | 0.00 | 0.00 |
| k__Bacteria;p__Actinobacteria;c__Actinobacteria;o__Actinomycetales;f__Microbacteriaceae;g__Agromyces | | 0.00 | 0.00 |
| k__Bacteria;p__Chloroflexi;c__Chloroflexi;o__Chloroflexales;Other;Other | | 0.00 | 0.00 |
| k__Bacteria;p__Chloroflexi;c__Ktedonobacteria;o__Thermogemmatisporales;f__Thermogemmatisporaceae;g__ | | 0.00 | 0.00 |
| k__Bacteria;p__Proteobacteria;c__Gammaproteobacteria;o__;f__;g__ | | 0.00 | 0.00 |
| k__Bacteria;p__Actinobacteria;c__Actinobacteria;o__Actinomycetales;f__Nocardioidaceae;g__Nocardioides | | 0.00 | 0.00 |
| k__Bacteria;p__Proteobacteria;c__Alphaproteobacteria;o__Rhizobiales;f__Rhizobiaceae;Other | | 0.00 | 0.00 |
| k__Bacteria;p__Actinobacteria;c__Actinobacteria;o__Actinomycetales;f__Intrasporangiaceae;Other | | 0.00 | 0.00 |
| k__Bacteria;p__Proteobacteria;c__Betaproteobacteria;o__Burkholderiales;f__Burkholderiaceae;g__ | | 0.00 | 0.00 |
| k__Bacteria;p__Firmicutes;c__Erysipelotrichi;o__Erysipelotrichales;f__Erysipelotrichaceae;g__[Eubacterium] | | 0.00 | 0.00 |
| k__Bacteria;p__Bacteroidetes;c__Bacteroidia;o__Bacteroidales;f__[Paraprevotellaceae];g__ | | 0.00 | 0.00 |
| k__Bacteria;p__Proteobacteria;c__Deltaproteobacteria;o__Syntrophobacterales;f__Syntrophaceae;g__Desulfomonile | | 0.00 | 0.00 |
| k__Bacteria;p__Proteobacteria;c__Alphaproteobacteria;o__Rhodobacterales;f__Rhodobacteraceae;g__Paracoccus | | 0.00 | 0.00 |
| k__Bacteria;p__Firmicutes;c__Clostridia;o__Clostridiales;f__[Tissierellaceae];g__Finegoldia | | 0.00 | 0.00 |
| k__Bacteria;p__Proteobacteria;c__Betaproteobacteria;o__Rhodocyclales;f__Rhodocyclaceae;g__Uliginosibacterium | | 0.00 | 0.00 |
| k__Bacteria;p__Proteobacteria;c__Deltaproteobacteria;o__Myxococcales;f__Myxococcaceae;g__Anaeromyxobacter | | 0.00 | 0.00 |
| k__Bacteria;p__Proteobacteria;c__Gammaproteobacteria;o__Pseudomonadales;f__Moraxellaceae;g__Acinetobacter | | 0.00 | 0.01 |
| k__Bacteria;p__Proteobacteria;c__Gammaproteobacteria;o__Pseudomonadales;f__Pseudomonadaceae;g__ | | 0.00 | 0.00 |
| k__Bacteria;p__Proteobacteria;c__Deltaproteobacteria;o__MIZ46;f__;g__ | | 0.00 | 0.03 |
| k__Bacteria;p__Firmicutes;c__Bacilli;o__Lactobacillales;f__Enterococcaceae;g__Vagococcus | | 0.00 | 0.00 |
| k__Bacteria;p__Actinobacteria;c__Acidimicrobiia;o__Acidimicrobiales;f__Iamiaceae;g__Iamia | | 0.00 | 0.00 |
| k__Bacteria;p__Actinobacteria;c__Actinobacteria;o__Actinomycetales;f__Intrasporangiaceae;g__ | | 0.00 | 0.00 |
| k__Bacteria;p__Actinobacteria;c__Actinobacteria;o__Actinomycetales;f__Micrococcaceae;Other | | 0.00 | 0.00 |
| k__Bacteria;p__Actinobacteria;c__Thermoleophilia;o__Solirubrobacterales;f__Solirubrobacteraceae;g__Solirubrobacter | | 0.00 | 0.00 |
| k__Bacteria;p__Chloroflexi;c__C0119;o__;f__;g__ | | 0.00 | 0.00 |
| k__Bacteria;p__Cyanobacteria;c__Oscillatoriophycideae;o__Chroococcales;Other;Other | | 0.00 | 0.00 |
| k__Bacteria;p__Cyanobacteria;c__Oscillatoriophycideae;o__Chroococcales;f__Microcystaceae;g__Microcystis | | 0.00 | 0.00 |
| k__Bacteria;p__Cyanobacteria;c__Synechococcophycideae;o__Pseudanabaenales;f__Pseudanabaenaceae;g__Pseudanabaena | | 0.00 | 0.00 |
| k__Bacteria;p__Proteobacteria;c__Betaproteobacteria;o__A21b;f__EB1003;g__ | | 0.00 | 0.00 |
| k__Bacteria;p__Proteobacteria;c__Gammaproteobacteria;o__Alteromonadales;f__125ds10;g__ | | 0.00 | 0.00 |
| k__Bacteria;p__Verrucomicrobia;c__[Pedosphaerae];o__[Pedosphaerales];f__R4-41B;g__ | | 0.00 | 0.00 |
| k__Bacteria;p__Proteobacteria;c__Alphaproteobacteria;o__Caulobacterales;f__Caulobacteraceae;g__Phenylobacterium | | 0.00 | 0.12 |
| k__Bacteria;p__Chlamydiae;c__Chlamydiia;o__Chlamydiales;f__Parachlamydiaceae;g__Candidatus Protochlamydia | | 0.00 | 0.00 |
| k__Bacteria;p__Firmicutes;c__Bacilli;o__Bacillales;f__Paenibacillaceae;g__Paenibacillus | | 0.00 | 0.00 |
| k__Bacteria;p__Proteobacteria;c__Alphaproteobacteria;o__Rickettsiales;f__Rickettsiaceae;g__ | | 0.00 | 0.00 |
| k__Bacteria;p__Proteobacteria;c__Betaproteobacteria;o__Burkholderiales;f__Oxalobacteraceae;g__Polynucleobacter | | 0.00 | 0.00 |
| k__Bacteria;p__Proteobacteria;c__Gammaproteobacteria;o__Chromatiales;f__Chromatiaceae;g__ | | 0.00 | 0.00 |
| k__Bacteria;p__Cyanobacteria;c__Oscillatoriophycideae;o__Chroococcales;f__Cyanobacteriaceae;g__ | | 0.00 | 0.00 |
| k__Bacteria;p__Cyanobacteria;c__Oscillatoriophycideae;o__Chroococcales;f__Gomphosphaeriaceae;Other | | 0.00 | 0.00 |
| k__Bacteria;p__Cyanobacteria;c__Oscillatoriophycideae;o__Chroococcales;f__Gomphosphaeriaceae;g__ | | 0.00 | 0.00 |
| k__Bacteria;p__Firmicutes;c__Clostridia;o__Clostridiales;f__Veillonellaceae;g__ | | 0.00 | 0.00 |
| k__Bacteria;p__TM7;c__TM7-3;o__CW040;f__;g__ | | 0.00 | 0.00 |
| k__Bacteria;p__Proteobacteria;c__Gammaproteobacteria;Other;Other;Other | | 0.00 | 0.01 |
| k__Bacteria;p__Proteobacteria;c__Alphaproteobacteria;o__Sphingomonadales;f__Sphingomonadaceae;g__Sphingobium | | 0.00 | 0.00 |
| k__Bacteria;p__Proteobacteria;c__Gammaproteobacteria;o__Aeromonadales;f__Aeromonadaceae;g__Aeromonas | | 0.00 | 0.00 |
| k__Bacteria;p__Actinobacteria;c__Actinobacteria;o__Actinomycetales;f__Nocardiaceae;g__Nocardia | | 0.00 | 0.00 |
| k__Bacteria;p__Firmicutes;c__Clostridia;o__OPB54;f__;g__ | | 0.00 | 0.00 |
| k__Bacteria;p__Firmicutes;c__Clostridia;o__Clostridiales;f__[Mogibacteriaceae];g__ | | 0.00 | 0.00 |
| k__Bacteria;p__Cyanobacteria;c__ML635J-21;o__;f__;g__ | | 0.00 | 0.00 |
| k__Bacteria;p__Cyanobacteria;c__Synechococcophycideae;o__Synechococcales;f__Acaryochloridaceae;g__Acaryochloris | | 0.00 | 0.00 |
| k__Bacteria;p__Planctomycetes;c__Phycisphaerae;o__Phycisphaerales;f__;g__ | | 0.00 | 0.00 |
| k__Bacteria;p__Proteobacteria;c__Deltaproteobacteria;o__Desulfobacterales;f__Desulfobacteraceae;g__Desulfococcus | | 0.00 | 0.00 |
| k__Bacteria;p__Proteobacteria;c__Gammaproteobacteria;o__HTCC2188;f__HTCC2089;g__ | | 0.00 | 0.00 |
| k__Bacteria;p__Verrucomicrobia;c__[Pedosphaerae];o__[Pedosphaerales];f__[Pedosphaeraceae];g__Pedosphaera | | 0.00 | 0.00 |
| k__Bacteria;p__Proteobacteria;c__Alphaproteobacteria;o__Caulobacterales;f__Caulobacteraceae;g__ | | 0.00 | 0.00 |
| k__Bacteria;p__Actinobacteria;c__Thermoleophilia;o__Gaiellales;f__;g__ | | 0.00 | 0.00 |
| k__Bacteria;p__Proteobacteria;c__Betaproteobacteria;o__Burkholderiales;f__Comamonadaceae;g__Methylibium | | 0.00 | 0.00 |
| k__Archaea;p__Euryarchaeota;c__Methanomicrobia;o__Methanosarcinales;f__Methanosaetaceae;g__Methanosaeta | | 0.00 | 0.00 |
| k__Bacteria;p__Firmicutes;c__Clostridia;o__Clostridiales;f__Clostridiaceae;g__Sarcina | | 0.00 | 0.00 |
| k__Bacteria;p__Proteobacteria;c__Betaproteobacteria;o__Rhodocyclales;f__Rhodocyclaceae;g__Candidatus Accumulibacter | | 0.00 | 0.00 |
| k__Bacteria;p__Proteobacteria;c__Epsilonproteobacteria;o__Campylobacterales;f__Campylobacteraceae;g__ | | 0.00 | 0.22 |
| k__Bacteria;p__Chlamydiae;c__Chlamydiia;o__Chlamydiales;f__Rhabdochlamydiaceae;g__Candidatus Rhabdochlamydia | | 0.00 | 0.03 |
| k__Bacteria;p__Proteobacteria;c__Alphaproteobacteria;o__Sphingomonadales;f__Sphingomonadaceae;g__Sphingomonas | | 0.00 | 0.01 |
| k__Bacteria;p__Actinobacteria;c__Actinobacteria;o__Actinomycetales;f__Micromonosporaceae;g__ | | 0.00 | 0.00 |
| k__Bacteria;p__TM7;c__TM7-3;o__I025;f__;g__ | | 0.00 | 0.00 |
| k__Bacteria;p__Tenericutes;c__RF3;o__ML615J-28;f__;g__ | | 0.00 | 0.00 |
| k__Bacteria;Other;Other;Other;Other;Other | | 0.00 | 0.00 |
| k__Bacteria;p__Chloroflexi;c__Ktedonobacteria;o__Ktedonobacterales;f__Ktedonobacteraceae;g__ | | 0.00 | 0.00 |
| k__Bacteria;p__Cyanobacteria;c__Chloroplast;o__Chlorophyta;Other;Other | | 0.00 | 0.00 |
| k__Bacteria;p__Gemmatimonadetes;c__Gemmatimonadetes;o__Ellin5290;f__;g__ | | 0.00 | 0.00 |
| k__Bacteria;p__Proteobacteria;c__Alphaproteobacteria;o__Rhizobiales;f__Beijerinckiaceae;g__Beijerinckia | | 0.00 | 0.00 |
| k__Bacteria;p__Proteobacteria;c__Alphaproteobacteria;o__Rhizobiales;f__Cohaesibacteraceae;g__Cohaesibacter | | 0.00 | 0.00 |
| k__Bacteria;p__Tenericutes;c__Mollicutes;o__RsaHF231;f__;g__ | | 0.00 | 0.00 |
| k__Bacteria;p__Chlamydiae;c__Chlamydiia;o__;f__;g__ | | 0.00 | 0.05 |
| k__Bacteria;p__Proteobacteria;c__Betaproteobacteria;o__Burkholderiales;f__Oxalobacteraceae;g__Ralstonia | | 0.00 | 0.01 |
| k__Bacteria;p__Proteobacteria;c__Gammaproteobacteria;o__Enterobacteriales;f__Enterobacteriaceae;g__Erwinia | | 0.00 | 0.01 |
| k__Bacteria;p__Bacteroidetes;c__Cytophagia;o__Cytophagales;f__Cytophagaceae;g__ | | 0.00 | 0.00 |
| k__Bacteria;p__Proteobacteria;c__Gammaproteobacteria;o__Vibrionales;f__Pseudoalteromonadaceae;g__Vibrio | | 0.00 | 0.00 |
| k__Bacteria;p__Actinobacteria;c__Actinobacteria;o__Actinomycetales;f__Cellulomonadaceae;g__Actinotalea | | 0.00 | 0.00 |
| k__Bacteria;p__Actinobacteria;c__Actinobacteria;o__Actinomycetales;f__Micrococcaceae;g__ | | 0.00 | 0.00 |
| k__Bacteria;p__Bacteroidetes;c__Bacteroidia;o__Bacteroidales;Other;Other | | 0.00 | 0.00 |
| k__Bacteria;p__Proteobacteria;c__Alphaproteobacteria;o__Rhizobiales;f__Bradyrhizobiaceae;g__Bradyrhizobium | | 0.00 | 0.00 |
| k__Bacteria;p__Proteobacteria;c__Gammaproteobacteria;o__Chromatiales;f__;g__ | | 0.00 | 0.00 |
| k__Bacteria;p__Acidobacteria;c__Acidobacteriia;o__Acidobacteriales;f__Koribacteraceae;g__Candidatus Koribacter | | 0.00 | 0.00 |
| k__Bacteria;p__Actinobacteria;c__Actinobacteria;o__Actinomycetales;f__Kineosporiaceae;Other | | 0.00 | 0.00 |
| k__Bacteria;p__Armatimonadetes;c__Armatimonadia;o__Armatimonadales;f__Armatimonadaceae;g__Armatimonas | | 0.00 | 0.00 |
| k__Bacteria;p__Chloroflexi;c__TK10;o__AKYG885;f__5B-12;g__ | | 0.00 | 0.00 |
| k__Bacteria;p__Cyanobacteria;c__Oscillatoriophycideae;o__Chroococcales;f__Spirulinaceae;g__Spirulina | | 0.00 | 0.00 |
| k__Bacteria;p__Firmicutes;c__Clostridia;o__Clostridiales;f__Gracilibacteraceae;g__Gracilibacter | | 0.00 | 0.00 |
| k__Bacteria;p__Firmicutes;c__Clostridia;o__Clostridiales;f__Peptostreptococcaceae;g__[Clostridium] | | 0.00 | 0.00 |
| k__Bacteria;p__Firmicutes;c__Clostridia;o__Clostridiales;f__[Mogibacteriaceae];g__Anaerovorax | | 0.00 | 0.00 |
| k__Bacteria;p__Proteobacteria;c__Alphaproteobacteria;o__Rhodospirillales;f__Rhodospirillaceae;g__Azospirillum | | 0.00 | 0.00 |
| k__Bacteria;p__Proteobacteria;c__Betaproteobacteria;o__Burkholderiales;f__Comamonadaceae;g__Rubrivivax | | 0.00 | 0.00 |
| k__Bacteria;p__Proteobacteria;c__Betaproteobacteria;o__Ellin6067;f__;g__ | | 0.00 | 0.00 |
| k__Bacteria;p__Proteobacteria;c__Deltaproteobacteria;o__Syntrophobacterales;f__Syntrophobacteraceae;g__Syntrophobacter | | 0.00 | 0.00 |
| k__Bacteria;p__Proteobacteria;c__Alphaproteobacteria;o__Rhizobiales;f__Rhizobiaceae;g__Kaistia | | 0.00 | 0.22 |
| k__Bacteria;p__Chlamydiae;c__Chlamydiia;o__Chlamydiales;f__Parachlamydiaceae;Other | | 0.00 | 0.02 |
| k__Bacteria;p__Firmicutes;c__Bacilli;o__Bacillales;f__Staphylococcaceae;g__Staphylococcus | | 0.00 | 0.01 |
| k__Bacteria;p__Actinobacteria;c__Actinobacteria;o__Actinomycetales;f__Corynebacteriaceae;g__Corynebacterium | | 0.00 | 0.01 |
| k__Bacteria;p__Actinobacteria;c__Actinobacteria;o__Actinomycetales;f__Frankiaceae;g__ | | 0.00 | 0.00 |
| k__Bacteria;p__Proteobacteria;c__Betaproteobacteria;o__Burkholderiales;f__Oxalobacteraceae;g__ | | 0.00 | 0.00 |
| k__Bacteria;p__Actinobacteria;c__Actinobacteria;o__Actinomycetales;f__Streptomycetaceae;g__Streptomyces | | 0.00 | 0.00 |
| k__Bacteria;p__Proteobacteria;c__Alphaproteobacteria;Other;Other;Other | | 0.00 | 0.00 |
| k__Bacteria;p__Proteobacteria;c__Alphaproteobacteria;o__Rhizobiales;f__Beijerinckiaceae;g__Chelatococcus | | 0.00 | 0.00 |
| k__Bacteria;p__Proteobacteria;c__Alphaproteobacteria;o__Rhodobacterales;f__Rhodobacteraceae;g__Amaricoccus | | 0.00 | 0.00 |
| k__Archaea;p__Euryarchaeota;c__Methanomicrobia;o__Methanocellales;f__Methanocellaceae;g__Methanocella | | 0.00 | 0.00 |
| k__Bacteria;p__Acidobacteria;c__S035;o__;f__;g__ | | 0.00 | 0.00 |
| k__Bacteria;p__Actinobacteria;c__Actinobacteria;o__Actinomycetales;f__ACK-M1;g__ | | 0.00 | 0.00 |
| k__Bacteria;p__Actinobacteria;c__Actinobacteria;o__Actinomycetales;f__Micromonosporaceae;Other | | 0.00 | 0.00 |
| k__Bacteria;p__Bacteroidetes;c__[Saprospirae];o__[Saprospirales];f__;g__ | | 0.00 | 0.00 |
| k__Bacteria;p__Chloroflexi;c__Gitt-GS-136;o__;f__;g__ | | 0.00 | 0.00 |
| k__Bacteria;p__Chloroflexi;c__TK17;o__;f__;g__ | | 0.00 | 0.00 |
| k__Bacteria;p__Cyanobacteria;c__Oscillatoriophycideae;o__Oscillatoriales;f__Phormidiaceae;g__Microcoleus | | 0.00 | 0.00 |
| k__Bacteria;p__Firmicutes;c__Clostridia;o__Clostridiales;f__Lachnospiraceae;g__Lachnospira | | 0.00 | 0.00 |
| k__Bacteria;p__Firmicutes;c__Clostridia;o__Clostridiales;f__Peptococcaceae;g__Desulfosporosinus | | 0.00 | 0.00 |
| k__Bacteria;p__Firmicutes;c__Erysipelotrichi;o__Erysipelotrichales;f__Erysipelotrichaceae;g__Holdemania | | 0.00 | 0.00 |
| k__Bacteria;p__Gemmatimonadetes;c__Gemm-1;o__;f__;g__ | | 0.00 | 0.00 |
| k__Bacteria;p__Planctomycetes;c__Planctomycetia;o__Gemmatales;f__Isosphaeraceae;g__Nostocoida | | 0.00 | 0.00 |
| k__Bacteria;p__Proteobacteria;c__Gammaproteobacteria;o__Chromatiales;f__Chromatiaceae;g__Thiorhodococcus | | 0.00 | 0.00 |
| k__Bacteria;p__Proteobacteria;c__Gammaproteobacteria;o__Methylococcales;f__Methylococcaceae;g__ | | 0.00 | 0.00 |
| k__Bacteria;p__Proteobacteria;c__Gammaproteobacteria;o__Xanthomonadales;f__Sinobacteraceae;g__Steroidobacter | | 0.00 | 0.00 |
| k__Bacteria;p__Verrucomicrobia;c__Opitutae;o__[Cerasicoccales];f__[Cerasicoccaceae];g__ | | 0.00 | 0.01 |
| k__Bacteria;p__Proteobacteria;c__Gammaproteobacteria;o__Xanthomonadales;f__Xanthomonadaceae;Other | | 0.00 | 0.01 |
| k__Bacteria;p__Chloroflexi;c__;o__;f__;g__ | | 0.00 | 0.00 |
| k__Bacteria;p__Actinobacteria;c__Actinobacteria;o__Actinomycetales;f__Pseudonocardiaceae;g__Pseudonocardia | | 0.00 | 0.00 |
| k__Bacteria;p__Firmicutes;c__Bacilli;o__Bacillales;Other;Other | | 0.00 | 0.00 |
| k__Bacteria;p__Firmicutes;c__Bacilli;o__Bacillales;f__Bacillaceae;Other | | 0.00 | 0.00 |
| k__Bacteria;p__OD1;c__;o__;f__;g__ | | 0.00 | 0.00 |
| k__Bacteria;p__Firmicutes;c__Clostridia;o__Clostridiales;f__Christensenellaceae;g__ | | 0.00 | 0.00 |
| k__Bacteria;p__Actinobacteria;c__Actinobacteria;o__Actinomycetales;f__Pseudonocardiaceae;g__Actinomycetospora | | 0.00 | 0.00 |
| k__Bacteria;p__Firmicutes;c__Bacilli;o__Bacillales;f__[Exiguobacteraceae];g__Exiguobacterium | | 0.00 | 0.00 |
| k__Bacteria;p__Proteobacteria;c__Betaproteobacteria;o__Burkholderiales;f__Burkholderiaceae;g__Burkholderia | | 0.00 | 0.00 |
| k__Bacteria;p__Planctomycetes;c__Phycisphaerae;o__Pla1;f__;g__ | | 0.00 | 0.00 |
| k__Bacteria;p__Bacteroidetes;c__Flavobacteriia;o__Flavobacteriales;f__[Weeksellaceae];g__Chryseobacterium | | 0.00 | 0.00 |
| k__Bacteria;p__Verrucomicrobia;c__[Spartobacteria];o__[Chthoniobacterales];f__[Chthoniobacteraceae];g__heteroC45_4W | | 0.00 | 0.00 |
| k__Bacteria;p__Actinobacteria;c__Actinobacteria;o__Actinomycetales;f__Sporichthyaceae;g__Sporichthya | | 0.00 | 0.00 |
| k__Bacteria;p__Actinobacteria;c__Actinobacteria;o__Actinomycetales;f__Streptomycetaceae;Other | | 0.00 | 0.00 |
| k__Bacteria;p__Proteobacteria;c__Gammaproteobacteria;o__Aeromonadales;f__Aeromonadaceae;Other | | 0.00 | 0.00 |
| k__Bacteria;p__Chlamydiae;c__Chlamydiia;o__Chlamydiales;f__Chlamydiaceae;g__ | | 0.00 | 0.00 |
| k__Bacteria;p__Firmicutes;c__Clostridia;o__SHA-98;f__;g__ | | 0.00 | 0.00 |
| k__Bacteria;p__Acidobacteria;c__Acidobacteria-5;o__;f__;g__ | | 0.00 | 0.00 |
| k__Bacteria;p__Actinobacteria;c__Actinobacteria;o__Actinomycetales;f__Actinosynnemataceae;Other | | 0.00 | 0.00 |
| k__Bacteria;p__Bacteroidetes;c__[Saprospirae];o__[Saprospirales];f__Chitinophagaceae;g__Sediminibacterium | | 0.00 | 0.00 |
| k__Bacteria;p__Chloroflexi;c__Anaerolineae;Other;Other;Other | | 0.00 | 0.00 |
| k__Bacteria;p__Chloroflexi;c__TK10;o__B07_WMSP1;f__;g__ | | 0.00 | 0.00 |
| k__Bacteria;p__Cyanobacteria;c__Chloroplast;o__Euglenozoa;f__;g__ | | 0.00 | 0.00 |
| k__Bacteria;p__Proteobacteria;c__Gammaproteobacteria;o__Pasteurellales;f__Pasteurellaceae;g__Haemophilus | | 0.00 | 0.00 |
| k__Bacteria;p__Nitrospirae;c__Nitrospira;o__Nitrospirales;f__Nitrospiraceae;g__Nitrospira | | 0.00 | 0.29 |
| k__Bacteria;p__Firmicutes;c__Clostridia;o__Clostridiales;f__[Tissierellaceae];g__Sporanaerobacter | | 0.00 | 0.09 |
| k__Bacteria;p__Proteobacteria;c__Gammaproteobacteria;o__Xanthomonadales;f__Xanthomonadaceae;g__Stenotrophomonas | | 0.00 | 0.05 |
| k__Bacteria;p__Proteobacteria;c__Gammaproteobacteria;o__Enterobacteriales;f__Enterobacteriaceae;g__Enterobacter | | 0.00 | 0.02 |
| k__Bacteria;p__Proteobacteria;c__Alphaproteobacteria;o__Rhizobiales;f__Bartonellaceae;g__ | | 0.00 | 0.00 |
| k__Bacteria;p__Proteobacteria;c__Gammaproteobacteria;o__Enterobacteriales;f__Enterobacteriaceae;g__Serratia | | 0.00 | 0.00 |
| k__Bacteria;p__Proteobacteria;c__Gammaproteobacteria;o__Vibrionales;f__Vibrionaceae;g__ | | 0.00 | 0.00 |
| k__Bacteria;p__Planctomycetes;c__Phycisphaerae;o__CPla-3;f__;g__ | | 0.00 | 0.00 |
| k__Bacteria;p__Tenericutes;c__Mollicutes;o__;f__;g__ | | 0.00 | 0.00 |
| k__Bacteria;p__Proteobacteria;c__Alphaproteobacteria;o__Rhizobiales;f__Brucellaceae;Other | | 0.00 | 0.00 |
| k__Bacteria;p__Proteobacteria;c__Betaproteobacteria;o__Rhodocyclales;f__Rhodocyclaceae;g__ | | 0.00 | 0.00 |
| k__Bacteria;p__Tenericutes;c__Mollicutes;o__RF39;f__;g__ | | 0.00 | 0.00 |
| k__Bacteria;p__Acidobacteria;c__RB25;o__;f__;g__ | | 0.00 | 0.00 |
| k__Bacteria;p__Actinobacteria;c__Actinobacteria;o__Actinomycetales;f__Micrococcaceae;g__Arthrobacter | | 0.00 | 0.00 |
| k__Bacteria;p__Actinobacteria;c__Actinobacteria;o__Actinomycetales;f__Micromonosporaceae;g__Actinoplanes | | 0.00 | 0.00 |
| k__Bacteria;p__Actinobacteria;c__Actinobacteria;o__Actinomycetales;f__Propionibacteriaceae;g__Luteococcus | | 0.00 | 0.00 |
| k__Bacteria;p__Actinobacteria;c__Thermoleophilia;o__Solirubrobacterales;f__Conexibacteraceae;g__Conexibacter | | 0.00 | 0.00 |
| k__Bacteria;p__Bacteroidetes;c__Bacteroidia;o__Bacteroidales;f__Porphyromonadaceae;g__Porphyromonas | | 0.00 | 0.00 |
| k__Bacteria;p__Chlamydiae;c__Chlamydiia;o__Chlamydiales;f__Criblamydiaceae;Other | | 0.00 | 0.00 |
| k__Bacteria;p__Chloroflexi;c__Ktedonobacteria;o__JG30-KF-AS9;f__;g__ | | 0.00 | 0.00 |
| k__Bacteria;p__Cyanobacteria;c__Chloroplast;o__Chlorophyta;f__Trebouxiophyceae;g__ | | 0.00 | 0.00 |
| k__Bacteria;p__Cyanobacteria;c__Oscillatoriophycideae;o__Chroococcales;f__Xenococcaceae;g__ | | 0.00 | 0.00 |
| k__Bacteria;p__Cyanobacteria;c__Synechococcophycideae;o__Synechococcales;f__Synechococcaceae;g__Prochlorococcus | | 0.00 | 0.00 |
| k__Bacteria;p__Firmicutes;c__Clostridia;o__Clostridiales;f__Clostridiaceae;g__Oxobacter | | 0.00 | 0.00 |
| k__Bacteria;p__Firmicutes;c__Clostridia;o__Clostridiales;f__[Tissierellaceae];g__Anaerococcus | | 0.00 | 0.00 |
| k__Bacteria;p__Fusobacteria;c__Fusobacteriia;o__Fusobacteriales;f__Fusobacteriaceae;g__u114 | | 0.00 | 0.00 |
| k__Bacteria;p__Proteobacteria;c__Alphaproteobacteria;o__Rhizobiales;f__Aurantimonadaceae;Other | | 0.00 | 0.00 |
| k__Bacteria;p__Proteobacteria;c__Alphaproteobacteria;o__Sphingomonadales;f__Sphingomonadaceae;g__Zymomonas | | 0.00 | 0.00 |
| k__Bacteria;p__Proteobacteria;c__Betaproteobacteria;o__Burkholderiales;Other;Other | | 0.00 | 0.00 |
| k__Bacteria;p__Proteobacteria;c__Betaproteobacteria;o__Methylophilales;f__Methylophilaceae;g__ | | 0.00 | 0.00 |
| k__Bacteria;p__Proteobacteria;c__Betaproteobacteria;o__Neisseriales;f__Neisseriaceae;g__Chromobacterium | | 0.00 | 0.00 |
| k__Bacteria;p__Proteobacteria;c__Deltaproteobacteria;Other;Other;Other | | 0.00 | 0.00 |
| k__Bacteria;p__Proteobacteria;c__Deltaproteobacteria;o__Desulfovibrionales;f__Desulfovibrionaceae;g__Desulfovibrio | | 0.00 | 0.00 |
| k__Bacteria;p__Proteobacteria;c__Deltaproteobacteria;o__Myxococcales;f__Myxococcaceae;g__Myxococcus | | 0.00 | 0.00 |
| k__Bacteria;p__Proteobacteria;c__Deltaproteobacteria;o__Myxococcales;f__OM27;g__ | | 0.00 | 0.00 |
| k__Bacteria;p__Proteobacteria;c__Epsilonproteobacteria;o__Campylobacterales;f__Campylobacteraceae;g__Sulfurospirillum | | 0.00 | 0.00 |
| k__Bacteria;p__Proteobacteria;c__Gammaproteobacteria;o__Chromatiales;f__Ectothiorhodospiraceae;g__ | | 0.00 | 0.00 |
| k__Bacteria;p__Proteobacteria;c__TA18;o__CV90;f__;g__ | | 0.00 | 0.00 |
| k__Bacteria;p__SBR1093;c__VHS-B5-50;o__;f__;g__ | | 0.00 | 0.00 |
| k__Bacteria;p__Synergistetes;c__Synergistia;o__Synergistales;f__Synergistaceae;g__Candidatus Tammella | | 0.00 | 0.00 |
| k__Bacteria;p__Verrucomicrobia;c__[Spartobacteria];o__[Chthoniobacterales];f__[Chthoniobacteraceae];g__OR-59 | | 0.00 | 0.00 |
| k__Bacteria;p__Firmicutes;c__Clostridia;o__Clostridiales;f__Eubacteriaceae;g__Garciella | | 0.00 | 0.15 |
| k__Bacteria;p__TM7;c__;o__;f__;g__ | | 0.00 | 0.05 |
| k__Bacteria;p__Firmicutes;c__Bacilli;o__Bacillales;f__Planococcaceae;g__Lysinibacillus | | 0.00 | 0.01 |
| k__Bacteria;p__Spirochaetes;c__[Brevinematae];o__[Brevinematales];f__Brevinemataceae;g__Brevinema | | 0.00 | 0.01 |
| k__Bacteria;p__Firmicutes;c__Clostridia;o__Clostridiales;f__[Tissierellaceae];g__Peptoniphilus | | 0.00 | 0.01 |
| k__Bacteria;p__Actinobacteria;c__Actinobacteria;o__Actinomycetales;f__Sanguibacteraceae;g__Sanguibacter | | 0.00 | 0.00 |
| k__Bacteria;p__TM6;c__SBRH58;o__;f__;g__ | | 0.00 | 0.00 |
| k__Bacteria;p__Proteobacteria;c__Gammaproteobacteria;o__Vibrionales;f__Vibrionaceae;g__Photobacterium | | 0.00 | 0.00 |
| k__Bacteria;p__Firmicutes;c__Bacilli;o__Bacillales;f__Planococcaceae;Other | | 0.00 | 0.00 |
| k__Bacteria;p__Actinobacteria;c__Actinobacteria;o__Actinomycetales;f__Microbacteriaceae;g__Clavibacter | | 0.00 | 0.00 |
| k__Bacteria;p__Actinobacteria;c__Actinobacteria;o__Actinomycetales;f__Actinomycetaceae;g__ | | 0.00 | 0.00 |
| k__Bacteria;p__Firmicutes;c__Clostridia;o__Clostridiales;f__Clostridiaceae;g__Caloramator | | 0.00 | 0.00 |
| k__Bacteria;p__Firmicutes;c__Clostridia;o__Clostridiales;f__Peptostreptococcaceae;g__Tepidibacter | | 0.00 | 0.00 |
| k__Bacteria;p__Chlamydiae;c__Chlamydiia;o__Chlamydiales;Other;Other | | 0.00 | 0.00 |
| k__Bacteria;p__Firmicutes;c__Clostridia;o__Clostridiales;f__Peptococcaceae;g__rc4-4 | | 0.00 | 0.00 |
| k__Bacteria;p__Actinobacteria;c__Actinobacteria;o__Actinomycetales;f__Beutenbergiaceae;Other | | 0.00 | 0.00 |
| k__Bacteria;p__Proteobacteria;c__Gammaproteobacteria;o__Enterobacteriales;f__Enterobacteriaceae;g__Trabulsiella | | 0.00 | 0.00 |
| k__Archaea;p__Crenarchaeota;c__Thaumarchaeota;o__Nitrososphaerales;f__Nitrososphaeraceae;g__Candidatus Nitrososphaera | | 0.00 | 0.00 |
| k__Bacteria;p__AD3;c__ABS-6;o__;f__;g__ | | 0.00 | 0.00 |
| k__Bacteria;p__Acidobacteria;c__[Chloracidobacteria];o__RB41;f__;g__ | | 0.00 | 0.00 |
| k__Bacteria;p__Actinobacteria;c__Acidimicrobiia;o__Acidimicrobiales;f__AKIW874;g__ | | 0.00 | 0.00 |
| k__Bacteria;p__Actinobacteria;c__Actinobacteria;o__Actinomycetales;f__Actinosynnemataceae;g__Kutzneria | | 0.00 | 0.00 |
| k__Bacteria;p__Actinobacteria;c__Actinobacteria;o__Actinomycetales;f__Geodermatophilaceae;Other | | 0.00 | 0.00 |
| k__Bacteria;p__Actinobacteria;c__Actinobacteria;o__Actinomycetales;f__Microbacteriaceae;g__Agrococcus | | 0.00 | 0.00 |
| k__Bacteria;p__Actinobacteria;c__Actinobacteria;o__Actinomycetales;f__Nocardioidaceae;g__Kribbella | | 0.00 | 0.00 |
| k__Bacteria;p__Bacteroidetes;c__Sphingobacteriia;o__Sphingobacteriales;f__;g__ | | 0.00 | 0.00 |
| k__Bacteria;p__Chlamydiae;c__Chlamydiia;o__Chlamydiales;f__Simkaniaceae;Other | | 0.00 | 0.00 |
| k__Bacteria;p__Chloroflexi;c__Anaerolineae;o__A31;f__;g__ | | 0.00 | 0.00 |
| k__Bacteria;p__Chloroflexi;c__Anaerolineae;o__envOPS12;f__;g__ | | 0.00 | 0.00 |
| k__Bacteria;p__Chloroflexi;c__S085;o__;f__;g__ | | 0.00 | 0.00 |
| k__Bacteria;p__Chloroflexi;c__TK10;o__AKYG885;f__Dolo_23;g__ | | 0.00 | 0.00 |
| k__Bacteria;p__Cyanobacteria;c__Chloroplast;o__Chlorophyta;f__Chlamydomonadaceae;Other | | 0.00 | 0.00 |
| k__Bacteria;p__Cyanobacteria;c__Synechococcophycideae;o__Pseudanabaenales;f__;g__ | | 0.00 | 0.00 |
| k__Bacteria;p__Firmicutes;Other;Other;Other;Other | | 0.00 | 0.00 |
| k__Bacteria;p__Firmicutes;c__Bacilli;o__Bacillales;f__[Thermicanaceae];g__Thermicanus | | 0.00 | 0.00 |
| k__Bacteria;p__Firmicutes;c__Clostridia;o__Clostridiales;f__Ruminococcaceae;Other | | 0.00 | 0.00 |
| k__Bacteria;p__NC10;c__12-24;o__JH-WHS47;f__;g__ | | 0.00 | 0.00 |
| k__Bacteria;p__Planctomycetes;c__OM190;o__CL500-15;f__;g__ | | 0.00 | 0.00 |
| k__Bacteria;p__Planctomycetes;c__Planctomycetia;o__Gemmatales;f__Isosphaeraceae;g__Isosphaera | | 0.00 | 0.00 |
| k__Bacteria;p__Proteobacteria;c__Alphaproteobacteria;o__Caulobacterales;f__Caulobacteraceae;g__Asticcacaulis | | 0.00 | 0.00 |
| k__Bacteria;p__Proteobacteria;c__Alphaproteobacteria;o__Rhodospirillales;f__Rhodospirillaceae;g__Inquilinus | | 0.00 | 0.00 |
| k__Bacteria;p__Proteobacteria;c__Alphaproteobacteria;o__Rickettsiales;f__Anaplasmataceae;g__Neorickettsia | | 0.00 | 0.00 |
| k__Bacteria;p__Proteobacteria;c__Betaproteobacteria;o__Burkholderiales;f__Burkholderiaceae;Other | | 0.00 | 0.00 |
| k__Bacteria;p__Proteobacteria;c__Betaproteobacteria;o__Burkholderiales;f__Comamonadaceae;g__Hydrogenophaga | | 0.00 | 0.00 |
| k__Bacteria;p__Proteobacteria;c__Deltaproteobacteria;o__PB19;f__;g__ | | 0.00 | 0.00 |
| k__Bacteria;p__Proteobacteria;c__Gammaproteobacteria;o__Oceanospirillales;f__Halomonadaceae;g__ | | 0.00 | 0.00 |
| k__Bacteria;p__Proteobacteria;c__Gammaproteobacteria;o__Oceanospirillales;f__Oleiphilaceae;g__ | | 0.00 | 0.00 |
| k__Bacteria;p__Verrucomicrobia;c__Verrucomicrobiae;o__Verrucomicrobiales;f__Verrucomicrobiaceae;g__Verrucomicrobium | | 0.00 | 0.00 |
| k__Bacteria;p__Verrucomicrobia;c__[Pedosphaerae];o__[Pedosphaerales];Other;Other | | 0.00 | 0.00 |
| k__Bacteria;p__Firmicutes;c__Bacilli;o__Bacillales;f__Bacillaceae;g__Virgibacillus | | 0.00 | 0.20 |
| k__Bacteria;p__Firmicutes;c__Bacilli;o__Bacillales;f__Bacillaceae;g__Geobacillus | | 0.00 | 0.05 |
| k__Bacteria;p__OP11;c__OP11-4;o__;f__;g__ | | 0.00 | 0.01 |
| k__Bacteria;p__Proteobacteria;c__Gammaproteobacteria;o__Vibrionales;f__Vibrionaceae;g__Salinivibrio | | 0.00 | 0.01 |
| k__Bacteria;p__Proteobacteria;c__Gammaproteobacteria;o__Xanthomonadales;f__Xanthomonadaceae;g__Pseudoxanthomonas | | 0.00 | 0.01 |
| k__Bacteria;p__Firmicutes;c__Bacilli;o__Bacillales;f__Staphylococcaceae;g__Jeotgalicoccus | | 0.00 | 0.01 |
| k__Bacteria;p__Bacteroidetes;c__Flavobacteriia;o__Flavobacteriales;f__[Weeksellaceae];g__Cloacibacterium | | 0.00 | 0.01 |
| k__Bacteria;p__Firmicutes;c__Bacilli;o__Bacillales;f__Planococcaceae;g__Ureibacillus | | 0.00 | 0.01 |
| k__Bacteria;p__Firmicutes;c__Clostridia;o__Clostridiales;f__[Tissierellaceae];Other | | 0.00 | 0.01 |
| k__Bacteria;p__Firmicutes;c__Clostridia;o__Clostridiales;f__[Tissierellaceae];g__Tepidimicrobium | | 0.00 | 0.00 |
| k__Bacteria;p__Firmicutes;c__Bacilli;o__Lactobacillales;f__Leuconostocaceae;g__ | | 0.00 | 0.00 |
| k__Bacteria;p__Actinobacteria;c__Actinobacteria;o__Actinomycetales;f__Microbacteriaceae;g__Rathayibacter | | 0.00 | 0.00 |
| k__Bacteria;p__Proteobacteria;c__Gammaproteobacteria;o__Xanthomonadales;f__Xanthomonadaceae;g__Lysobacter | | 0.00 | 0.00 |
| k__Bacteria;p__TM6;Other;Other;Other;Other | | 0.00 | 0.00 |
| k__Bacteria;p__Actinobacteria;c__KIST-JJY010;o__;f__;g__ | | 0.00 | 0.00 |
| k__Bacteria;p__Firmicutes;c__Clostridia;o__Clostridiales;f__[Tissierellaceae];g__ | | 0.00 | 0.00 |
| k__Bacteria;p__Firmicutes;c__Bacilli;o__Lactobacillales;f__Lactobacillaceae;Other | | 0.00 | 0.00 |
| k__Bacteria;p__Firmicutes;c__Bacilli;o__Bacillales;f__Planococcaceae;g__Sporosarcina | | 0.00 | 0.00 |
| k__Bacteria;p__Chlamydiae;c__Chlamydiia;o__Chlamydiales;f__Parachlamydiaceae;g__ | | 0.00 | 0.00 |
| k__Bacteria;p__Chlamydiae;c__Chlamydiia;o__Chlamydiales;f__Waddliaceae;g__ | | 0.00 | 0.00 |
| k__Bacteria;p__Gemmatimonadetes;c__Gemmatimonadetes;o__Gemmatimonadales;f__;g__ | | 0.00 | 0.00 |
| k__Bacteria;p__Actinobacteria;c__Actinobacteria;o__Actinomycetales;f__Micrococcaceae;g__Micrococcus | | 0.00 | 0.00 |
| k__Bacteria;p__Firmicutes;c__Clostridia;o__Thermoanaerobacterales;f__;g__ | | 0.00 | 0.00 |
| k__Bacteria;p__Actinobacteria;c__Actinobacteria;o__Actinomycetales;f__Dermabacteraceae;g__Brachybacterium | | 0.00 | 0.00 |
| k__Bacteria;p__Actinobacteria;c__Actinobacteria;o__Actinomycetales;f__Brevibacteriaceae;g__Brevibacterium | | 0.00 | 0.00 |
| k__Bacteria;p__Firmicutes;c__Erysipelotrichi;o__Erysipelotrichales;f__Erysipelotrichaceae;g__Erysipelothrix | | 0.00 | 0.00 |
| k__Bacteria;p__Proteobacteria;c__Gammaproteobacteria;o__Vibrionales;f__Vibrionaceae;g__Aliivibrio | | 0.00 | 0.00 |
| k__Bacteria;p__Actinobacteria;c__Actinobacteria;o__Actinomycetales;f__Propionibacteriaceae;g__Propionibacterium | | 0.00 | 0.00 |
| k__Bacteria;p__Actinobacteria;c__Coriobacteriia;o__Coriobacteriales;f__Coriobacteriaceae;g__ | | 0.00 | 0.00 |
| k__Bacteria;p__Bacteroidetes;c__Cytophagia;o__Cytophagales;f__Cytophagaceae;g__Sporocytophaga | | 0.00 | 0.00 |
| k__Bacteria;p__Firmicutes;c__Bacilli;o__Bacillales;f__Planococcaceae;g__ | | 0.00 | 0.00 |
| k__Bacteria;p__Fusobacteria;c__Fusobacteriia;o__Fusobacteriales;f__Fusobacteriaceae;g__Psychrilyobacter | | 0.00 | 0.00 |
| k__Bacteria;p__Proteobacteria;c__Gammaproteobacteria;o__Alteromonadales;f__Psychromonadaceae;g__Psychromonas | | 0.00 | 0.00 |
| k__Bacteria;p__Proteobacteria;c__Alphaproteobacteria;o__Rhizobiales;f__Phyllobacteriaceae;g__Phyllobacterium | | 0.00 | 0.00 |
| k__Bacteria;p__Proteobacteria;c__Gammaproteobacteria;o__Xanthomonadales;f__Xanthomonadaceae;g__Luteimonas | | 0.00 | 0.00 |
| k__Bacteria;p__Proteobacteria;c__Gammaproteobacteria;o__Pseudomonadales;f__Moraxellaceae;g__Psychrobacter | | 0.00 | 0.00 |
| k__Bacteria;p__TM6;c__SJA-4;o__S1198;f__;g__ | | 0.00 | 0.00 |
| k__Bacteria;p__Bacteroidetes;c__Flavobacteriia;o__Flavobacteriales;f__Flavobacteriaceae;g__ | | 0.00 | 0.00 |
| k__Bacteria;p__Proteobacteria;c__Alphaproteobacteria;o__Sphingomonadales;f__Erythrobacteraceae;g__ | | 0.00 | 0.00 |
| k__Bacteria;p__MVP-21;c__;o__;f__;g__ | | 0.00 | 0.00 |
| k__Bacteria;p__Actinobacteria;c__Actinobacteria;o__Actinomycetales;f__Micrococcaceae;g__Kocuria | | 0.00 | 0.00 |
| k__Bacteria;p__Chloroflexi;c__Chloroflexi;o__AKIW781;f__;g__ | | 0.00 | 0.00 |
| k__Bacteria;p__Tenericutes;c__Mollicutes;o__Mycoplasmatales;f__Mycoplasmataceae;g__ | | 0.00 | 0.00 |
| k__Bacteria;p__Bacteroidetes;c__Cytophagia;o__Cytophagales;f__Cytophagaceae;g__Spirosoma | | 0.00 | 0.00 |
| k__Bacteria;p__Firmicutes;c__Bacilli;o__Bacillales;f__Staphylococcaceae;g__Salinicoccus | | 0.00 | 0.00 |
| k__Bacteria;p__Firmicutes;c__Bacilli;o__Lactobacillales;f__Leuconostocaceae;g__Weissella | | 0.00 | 0.00 |
| k__Bacteria;p__Bacteroidetes;c__Cytophagia;o__Cytophagales;f__Cytophagaceae;g__Emticicia | | 0.00 | 0.00 |
| k__Bacteria;p__Chloroflexi;c__Ktedonobacteria;o__;f__;g__ | | 0.00 | 0.00 |
| k__Bacteria;p__Firmicutes;c__Bacilli;o__Bacillales;f__Planococcaceae;g__Kurthia | | 0.00 | 0.00 |
| k__Bacteria;p__Firmicutes;c__Bacilli;o__Lactobacillales;f__Aerococcaceae;g__Facklamia | | 0.00 | 0.00 |
| k__Bacteria;p__Proteobacteria;c__Alphaproteobacteria;o__Caulobacterales;f__Caulobacteraceae;g__Brevundimonas | | 0.00 | 0.00 |
| k__Bacteria;p__Proteobacteria;c__Alphaproteobacteria;o__Sphingomonadales;f__Sphingomonadaceae;g__Sphingopyxis | | 0.00 | 0.00 |
| k__Bacteria;p__TM6;c__SJA-4;o__YJF2-48;f__;g__ | | 0.00 | 0.00 |
| k__Bacteria;p__Armatimonadetes;c__[Fimbriimonadia];o__[Fimbriimonadales];f__[Fimbriimonadaceae];g__Fimbriimonas | | 0.00 | 0.00 |
| k__Bacteria;p__Firmicutes;c__Bacilli;o__Bacillales;f__Bacillaceae;g__Lentibacillus | | 0.00 | 0.00 |
| k__Bacteria;p__Proteobacteria;c__Alphaproteobacteria;o__Rhizobiales;f__Methylocystaceae;g__Methylopila | | 0.00 | 0.00 |
| k__Bacteria;p__Proteobacteria;c__Gammaproteobacteria;o__Legionellales;f__Coxiellaceae;g__Coxiella | | 0.00 | 0.00 |
| k__Bacteria;p__Proteobacteria;c__Gammaproteobacteria;o__Xanthomonadales;f__Xanthomonadaceae;g__Xanthomonas | | 0.00 | 0.00 |
| k__Bacteria;p__Actinobacteria;c__Actinobacteria;o__Actinomycetales;f__Bogoriellaceae;g__Georgenia | | 0.00 | 0.00 |
| k__Bacteria;p__Actinobacteria;c__Actinobacteria;o__Actinomycetales;f__Gordoniaceae;g__Gordonia | | 0.00 | 0.00 |
| k__Bacteria;p__Actinobacteria;c__Actinobacteria;o__Actinomycetales;f__Pseudonocardiaceae;g__ | | 0.00 | 0.00 |
| k__Bacteria;p__Cyanobacteria;c__Chloroplast;o__CAB-I;f__;g__ | | 0.00 | 0.00 |
| k__Bacteria;p__Firmicutes;c__Bacilli;o__Bacillales;f__Paenibacillaceae;g__Aneurinibacillus | | 0.00 | 0.00 |
| k__Bacteria;p__Firmicutes;c__Bacilli;o__Lactobacillales;f__Carnobacteriaceae;g__Carnobacterium | | 0.00 | 0.00 |
| k__Bacteria;p__Fusobacteria;c__Fusobacteriia;o__Fusobacteriales;f__Fusobacteriaceae;g__Propionigenium | | 0.00 | 0.00 |
| k__Bacteria;p__Actinobacteria;c__Actinobacteria;o__Actinomycetales;f__Microbacteriaceae;g__Mycetocola | | 0.00 | 0.00 |
| k__Bacteria;p__Actinobacteria;c__Actinobacteria;o__Actinomycetales;f__Micrococcaceae;g__Rothia | | 0.00 | 0.00 |
| k__Bacteria;p__Firmicutes;c__Bacilli;o__Bacillales;f__Paenibacillaceae;g__Brevibacillus | | 0.00 | 0.00 |
| k__Bacteria;p__Firmicutes;c__Bacilli;o__Lactobacillales;f__Leuconostocaceae;g__Leuconostoc | | 0.00 | 0.00 |
| k__Bacteria;p__Firmicutes;c__Clostridia;o__Clostridiales;f__Caldicoprobacteraceae;g__Caldicoprobacter | | 0.00 | 0.00 |
| k__Bacteria;p__Firmicutes;c__Clostridia;o__Clostridiales;f__Gracilibacteraceae;g__ | | 0.00 | 0.00 |
| k__Bacteria;p__OP11;c__WCHB1-64;o__;f__;g__ | | 0.00 | 0.00 |
| k__Bacteria;p__Proteobacteria;c__Gammaproteobacteria;o__Enterobacteriales;f__Enterobacteriaceae;g__Proteus | | 0.00 | 0.00 |
| k__Bacteria;p__Spirochaetes;c__[Brevinematae];o__[Brevinematales];f__Brevinemataceae;g__ | | 0.00 | 0.00 |
| k__Bacteria;p__Actinobacteria;c__Actinobacteria;o__Actinomycetales;f__Microbacteriaceae;g__Curtobacterium | | 0.00 | 0.00 |
| k__Bacteria;p__Bacteroidetes;c__Flavobacteriia;o__Flavobacteriales;f__[Weeksellaceae];g__ | | 0.00 | 0.00 |
| k__Bacteria;p__Bacteroidetes;c__Sphingobacteriia;o__Sphingobacteriales;f__Sphingobacteriaceae;g__Sphingobacterium | | 0.00 | 0.00 |
| k__Bacteria;p__Proteobacteria;c__Betaproteobacteria;o__Burkholderiales;f__;g__ | | 0.00 | 0.00 |
| k__Bacteria;p__Proteobacteria;c__Betaproteobacteria;o__Neisseriales;f__Neisseriaceae;g__Neisseria | | 0.00 | 0.00 |
| k__Bacteria;p__Proteobacteria;c__Deltaproteobacteria;o__Bdellovibrionales;f__Bacteriovoracaceae;g__ | | 0.00 | 0.00 |
| k__Bacteria;p__Proteobacteria;c__Gammaproteobacteria;o__Aeromonadales;f__;g__ | | 0.00 | 0.00 |
| k__Bacteria;p__Proteobacteria;c__Gammaproteobacteria;o__Xanthomonadales;f__Xanthomonadaceae;g__Dokdonella | | 0.00 | 0.00 |
| k__Bacteria;p__Spirochaetes;c__Spirochaetes;o__Sphaerochaetales;f__Sphaerochaetaceae;g__ | | 0.00 | 0.00 |
| k__Bacteria;p__Actinobacteria;c__Actinobacteria;o__Actinomycetales;f__Nocardiaceae;g__ | | 0.00 | 0.00 |
| k__Bacteria;p__Actinobacteria;c__Actinobacteria;o__Actinomycetales;f__Sporichthyaceae;g__ | | 0.00 | 0.00 |
| k__Bacteria;p__Actinobacteria;c__Actinobacteria;o__Actinomycetales;f__Thermomonosporaceae;g__Actinomadura | | 0.00 | 0.00 |
| k__Bacteria;p__Bacteroidetes;c__Cytophagia;o__Cytophagales;f__Cytophagaceae;g__Hymenobacter | | 0.00 | 0.00 |
| k__Bacteria;p__Bacteroidetes;c__[Saprospirae];o__[Saprospirales];f__Chitinophagaceae;g__Niabella | | 0.00 | 0.00 |
| k__Bacteria;p__Firmicutes;c__Bacilli;o__Lactobacillales;f__Carnobacteriaceae;g__Granulicatella | | 0.00 | 0.00 |
| k__Bacteria;p__Firmicutes;c__Clostridia;o__Clostridiales;f__Veillonellaceae;g__Sporomusa | | 0.00 | 0.00 |
| k__Bacteria;p__Firmicutes;c__Erysipelotrichi;o__Erysipelotrichales;f__Erysipelotrichaceae;g__cc_115 | | 0.00 | 0.00 |
| k__Bacteria;p__Proteobacteria;c__Gammaproteobacteria;o__Vibrionales;f__Vibrionaceae;g__Enterovibrio | | 0.00 | 0.00 |
| k__Bacteria;p__TM7;c__MJK10;o__;f__;g__ | | 0.00 | 0.00 |
| k__Bacteria;p__Actinobacteria;c__Actinobacteria;o__Actinomycetales;f__Cellulomonadaceae;Other | | 0.00 | 0.00 |
| k__Bacteria;p__Firmicutes;c__Bacilli;o__Bacillales;f__Paenibacillaceae;g__ | | 0.00 | 0.00 |
| k__Bacteria;p__Firmicutes;c__Bacilli;o__Gemellales;f__Gemellaceae;g__ | | 0.00 | 0.00 |
| k__Bacteria;p__Firmicutes;c__Erysipelotrichi;o__Erysipelotrichales;f__Erysipelotrichaceae;g__Bulleidia | | 0.00 | 0.00 |
| k__Bacteria;p__OD1;c__ZB2;o__;f__;g__ | | 0.00 | 0.00 |
| k__Bacteria;p__Proteobacteria;c__Alphaproteobacteria;o__Rhizobiales;f__Phyllobacteriaceae;g__Nitratireductor | | 0.00 | 0.00 |
| k__Bacteria;p__Proteobacteria;c__Betaproteobacteria;o__Burkholderiales;f__Alcaligenaceae;g__Alcaligenes | | 0.00 | 0.00 |
| k__Bacteria;p__Proteobacteria;c__Gammaproteobacteria;o__Alteromonadales;f__Alteromonadaceae;g__Marinobacter | | 0.00 | 0.00 |
| k__Bacteria;p__Proteobacteria;c__Gammaproteobacteria;o__Chromatiales;Other;Other | | 0.00 | 0.00 |
| k__Bacteria;p__Proteobacteria;c__Gammaproteobacteria;o__Enterobacteriales;f__Enterobacteriaceae;g__Klebsiella | | 0.00 | 0.00 |
| k__Bacteria;p__Proteobacteria;c__Gammaproteobacteria;o__Oceanospirillales;f__Halomonadaceae;g__Halomonas | | 0.00 | 0.00 |
| k__Bacteria;p__Actinobacteria;c__Actinobacteria;o__Actinomycetales;f__Actinosynnemataceae;g__Lentzea | | 0.00 | 0.00 |
| k__Bacteria;p__Actinobacteria;c__Actinobacteria;o__Actinomycetales;f__Dietziaceae;g__Dietzia | | 0.00 | 0.00 |
| k__Bacteria;p__Actinobacteria;c__Actinobacteria;o__Actinomycetales;f__Microbacteriaceae;g__Candidatus Rhodoluna | | 0.00 | 0.00 |
| k__Bacteria;p__Actinobacteria;c__Actinobacteria;o__Actinomycetales;f__Propionibacteriaceae;Other | | 0.00 | 0.00 |
| k__Bacteria;p__Actinobacteria;c__Actinobacteria;o__Actinomycetales;f__Pseudonocardiaceae;g__Amycolatopsis | | 0.00 | 0.00 |
| k__Bacteria;p__Actinobacteria;c__Actinobacteria;o__Actinomycetales;f__Streptomycetaceae;g__ | | 0.00 | 0.00 |
| k__Bacteria;p__Actinobacteria;c__Actinobacteria;o__Bifidobacteriales;f__Bifidobacteriaceae;Other | | 0.00 | 0.00 |
| k__Bacteria;p__Chloroflexi;c__Thermomicrobia;o__AKYG1722;f__;g__ | | 0.00 | 0.00 |
| k__Bacteria;p__Firmicutes;c__Bacilli;Other;Other;Other | | 0.00 | 0.00 |
| k__Bacteria;p__Firmicutes;c__Bacilli;o__Bacillales;f__Bacillaceae;g__Anaerobacillus | | 0.00 | 0.00 |
| k__Bacteria;p__Firmicutes;c__Bacilli;o__Bacillales;f__Planococcaceae;g__Paenisporosarcina | | 0.00 | 0.00 |
| k__Bacteria;p__Firmicutes;c__Bacilli;o__Bacillales;f__Planococcaceae;g__Rummeliibacillus | | 0.00 | 0.00 |
| k__Bacteria;p__Firmicutes;c__Bacilli;o__Bacillales;f__Staphylococcaceae;g__Macrococcus | | 0.00 | 0.00 |
| k__Bacteria;p__Firmicutes;c__Bacilli;o__Lactobacillales;Other;Other | | 0.00 | 0.00 |
| k__Bacteria;p__Firmicutes;c__Bacilli;o__Lactobacillales;f__Carnobacteriaceae;Other | | 0.00 | 0.00 |
| k__Bacteria;p__Firmicutes;c__Bacilli;o__Lactobacillales;f__Lactobacillaceae;g__Pediococcus | | 0.00 | 0.00 |
| k__Bacteria;p__Firmicutes;c__Clostridia;o__Clostridiales;f__Clostridiaceae;g__Alkaliphilus | | 0.00 | 0.00 |
| k__Bacteria;p__Firmicutes;c__Clostridia;o__Clostridiales;f__Lachnospiraceae;g__Roseburia | | 0.00 | 0.00 |
| k__Bacteria;p__Proteobacteria;c__Alphaproteobacteria;o__Caulobacterales;f__Caulobacteraceae;Other | | 0.00 | 0.00 |
| k__Bacteria;p__Proteobacteria;c__Alphaproteobacteria;o__Caulobacterales;f__Caulobacteraceae;g__Caulobacter | | 0.00 | 0.00 |
| k__Bacteria;p__Proteobacteria;c__Alphaproteobacteria;o__Rhizobiales;f__Bradyrhizobiaceae;g__Afipia | | 0.00 | 0.00 |
| k__Bacteria;p__Proteobacteria;c__Alphaproteobacteria;o__Rhizobiales;f__Rhizobiaceae;g__Shinella | | 0.00 | 0.00 |
| k__Bacteria;p__Proteobacteria;c__Alphaproteobacteria;o__Rhodobacterales;Other;Other | | 0.00 | 0.00 |
| k__Bacteria;p__Proteobacteria;c__Alphaproteobacteria;o__Rhodospirillales;f__Rhodospirillaceae;Other | | 0.00 | 0.00 |
| k__Bacteria;p__Proteobacteria;c__Alphaproteobacteria;o__Sphingomonadales;Other;Other | | 0.00 | 0.00 |
| k__Bacteria;p__Proteobacteria;c__Betaproteobacteria;o__Burkholderiales;f__Burkholderiaceae;g__Lautropia | | 0.00 | 0.00 |
| k__Bacteria;p__Proteobacteria;c__Betaproteobacteria;o__Burkholderiales;f__Comamonadaceae;g__Comamonas | | 0.00 | 0.00 |
| k__Bacteria;p__Proteobacteria;c__Betaproteobacteria;o__Burkholderiales;f__Comamonadaceae;g__Roseateles | | 0.00 | 0.00 |
| k__Bacteria;p__Proteobacteria;c__Gammaproteobacteria;o__Aeromonadales;Other;Other | | 0.00 | 0.00 |
| k__Bacteria;p__Proteobacteria;c__Gammaproteobacteria;o__Alteromonadales;f__Alteromonadaceae;g__Cellvibrio | | 0.00 | 0.00 |
| k__Bacteria;p__Proteobacteria;c__Gammaproteobacteria;o__Oceanospirillales;f__Alcanivoracaceae;g__Alcanivorax | | 0.00 | 0.00 |
| k__Bacteria;p__Proteobacteria;c__Gammaproteobacteria;o__Xanthomonadales;f__Xanthomonadaceae;g__Thermomonas | | 0.00 | 0.00 |
| k__Bacteria;p__Spirochaetes;c__Spirochaetes;o__Sphaerochaetales;f__Sphaerochaetaceae;Other | | 0.00 | 0.00 |
| k__Bacteria;p__TM6;c__;o__;f__;g__ | | 0.00 | 0.00 |
