# Supplementary material for: Comparative study of gut microbiota in wild and captive Malaysian Mahseer (Tor tambroides)
Source: Microbiologyopen. 2018 Oct 23;8(5):e00734. doi: 10.1002/mbo3.734 (PMC6528585; doi:10.1002/mbo3.734)
Supplement: Supplementary file 2 [file MBO3-8-e00734-s002.docx]

**SUPPLEMENTARY TABLE B: Function Predictions from PICRUSt**

| # Constructed from biom file | Mean | |  |
| --- | --- | --- | --- |
| KEGG_Pathways | Wild | Captive | P-value |
| Metabolism; Glycan Biosynthesis and Metabolism; Lipopolysaccharide biosynthesis | 364241.02 | 364485.35 | 0.499 |
| Metabolism; Xenobiotics Biodegradation and Metabolism; Chlorocyclohexane and chlorobenzene degradation | 43722.97 | 43774.63 | 0.499 |
| Metabolism; Energy Metabolism; Photosynthesis - antenna proteins | 12330.79 | 12439.27 | 0.497 |
| Metabolism; Carbohydrate Metabolism; Glycolysis / Gluconeogenesis | 1169964.98 | 1172655.88 | 0.485 |
| Metabolism; Xenobiotics Biodegradation and Metabolism; 1,1,1-Trichloro-2,2-bis(4-chlorophenyl)ethane (DDT) degradation | 423.97 | 416.01 | 0.484 |
| Metabolism; Metabolism of Terpenoids and Polyketides; Limonene and pinene degradation | 207101.20 | 210614.28 | 0.481 |
| Metabolism; Biosynthesis of Other Secondary Metabolites; Tropane, piperidine and pyridine alkaloid biosynthesis | 132401.95 | 132648.79 | 0.479 |
| Metabolism; Energy Metabolism; Nitrogen metabolism | 826818.40 | 821955.07 | 0.473 |
| Organismal Systems; Digestive System; Protein digestion and absorption | 4048.08 | 4346.36 | 0.463 |
| Metabolism; Biosynthesis of Other Secondary Metabolites; Penicillin and cephalosporin biosynthesis | 41103.06 | 39913.90 | 0.463 |
| Human Diseases; Immune System Diseases; Primary immunodeficiency | 47369.05 | 46091.09 | 0.462 |
| Metabolism; Xenobiotics Biodegradation and Metabolism; Atrazine degradation | 32294.66 | 34799.29 | 0.458 |
| Metabolism; Xenobiotics Biodegradation and Metabolism; Polycyclic aromatic hydrocarbon degradation | 96366.64 | 99746.32 | 0.458 |
| Genetic Information Processing; Replication and Repair; Non-homologous end-joining | 21377.59 | 23099.94 | 0.451 |
| Human Diseases; Neurodegenerative Diseases; Huntington's disease | 64034.75 | 66490.67 | 0.449 |
| Environmental Information Processing; Signal Transduction; Calcium signaling pathway | 751.24 | 861.68 | 0.448 |
| Human Diseases; Cancers; Pathways in cancer | 51587.80 | 50094.76 | 0.447 |
| Metabolism; Carbohydrate Metabolism; Glyoxylate and dicarboxylate metabolism | 646282.04 | 656590.95 | 0.446 |
| Environmental Information Processing; Signal Transduction; Two-component system | 2150173.65 | 2126458.74 | 0.446 |
| Human Diseases; Immune System Diseases; Systemic lupus erythematosus | 395.08 | 344.02 | 0.440 |
| Metabolism; Lipid Metabolism; Fatty acid metabolism | 517828.94 | 504724.55 | 0.425 |
| Metabolism; Carbohydrate Metabolism; Citrate cycle (TCA cycle) | 940709.04 | 903212.88 | 0.422 |
| Metabolism; Glycan Biosynthesis and Metabolism; Other glycan degradation | 82659.91 | 84952.95 | 0.421 |
| Metabolism; Amino Acid Metabolism; Lysine degradation | 288290.47 | 303958.36 | 0.421 |
| Metabolism; Amino Acid Metabolism; Cysteine and methionine metabolism | 973664.58 | 957767.45 | 0.412 |
| Environmental Information Processing; Signaling Molecules and Interaction; Ion channels | 63167.64 | 56436.89 | 0.410 |
| Unclassified; Metabolism; Metabolism of cofactors and vitamins | 214131.18 | 218623.22 | 0.410 |
| Metabolism; Amino Acid Metabolism; Valine, leucine and isoleucine degradation | 557913.40 | 528618.00 | 0.410 |
| Metabolism; Biosynthesis of Other Secondary Metabolites; Clavulanic acid biosynthesis | 7.67 | 9.80 | 0.409 |
| Metabolism; Metabolism of Cofactors and Vitamins; Vitamin B6 metabolism | 191109.66 | 193827.36 | 0.409 |
| Organismal Systems; Excretory System; Proximal tubule bicarbonate reclamation | 37524.42 | 38473.45 | 0.407 |
| Metabolism; Biosynthesis of Other Secondary Metabolites; Isoquinoline alkaloid biosynthesis | 81652.51 | 79499.19 | 0.407 |
| Metabolism; Amino Acid Metabolism; Arginine and proline metabolism | 1143115.96 | 1165219.73 | 0.406 |
| Human Diseases; Infectious Diseases; Amoebiasis | 4862.88 | 6060.63 | 0.405 |
| Metabolism; Biosynthesis of Other Secondary Metabolites; beta-Lactam resistance | 24086.56 | 25611.22 | 0.404 |
| Genetic Information Processing; Replication and Repair; Chromosome | 1304588.56 | 1282329.66 | 0.404 |
| Cellular Processes; Transport and Catabolism; Lysosome | 40132.29 | 37009.29 | 0.404 |
| Metabolism; Enzyme Families; Peptidases | 1619262.94 | 1603294.09 | 0.404 |
| Metabolism; Energy Metabolism; Carbon fixation in photosynthetic organisms | 563686.01 | 560349.34 | 0.402 |
| Metabolism; Metabolism of Other Amino Acids; D-Alanine metabolism | 84392.09 | 83173.32 | 0.400 |
| Organismal Systems; Nervous System; Glutamatergic synapse | 85421.13 | 82523.00 | 0.399 |
| Metabolism; Lipid Metabolism; Steroid hormone biosynthesis | 15943.69 | 18410.62 | 0.398 |
| Genetic Information Processing; Translation; Ribosome Biogenesis | 1246678.04 | 1207896.27 | 0.386 |
| Unclassified; Cellular Processes and Signaling; Signal transduction mechanisms | 437383.21 | 445134.81 | 0.379 |
| Cellular Processes; Transport and Catabolism; Endocytosis | 67.15 | 97.32 | 0.377 |
| Organismal Systems; Endocrine System; GnRH signaling pathway | 67.15 | 97.32 | 0.377 |
| Organismal Systems; Immune System; Fc gamma R-mediated phagocytosis | 67.15 | 97.32 | 0.377 |
| Metabolism; Enzyme Families; Cytochrome P450 | 76.20 | 113.07 | 0.374 |
| Metabolism; Metabolism of Cofactors and Vitamins; Nicotinate and nicotinamide metabolism | 446618.27 | 433617.91 | 0.371 |
| Organismal Systems; Immune System; Hematopoietic cell lineage | 67.67 | 100.76 | 0.370 |
| Metabolism; Metabolism of Other Amino Acids; D-Arginine and D-ornithine metabolism | 4274.31 | 3032.38 | 0.368 |
| Metabolism; Xenobiotics Biodegradation and Metabolism; Drug metabolism - other enzymes | 295475.24 | 286375.61 | 0.367 |
| Cellular Processes; Cell Growth and Death; Meiosis - yeast | 25556.16 | 17530.26 | 0.364 |
| Genetic Information Processing; Translation; RNA transport | 117510.40 | 107954.97 | 0.360 |
| Metabolism; Metabolism of Other Amino Acids; Taurine and hypotaurine metabolism | 133646.23 | 138631.38 | 0.360 |
| Metabolism; Xenobiotics Biodegradation and Metabolism; Metabolism of xenobiotics by cytochrome P450 | 103178.62 | 116711.65 | 0.352 |
| Metabolism; Metabolism of Other Amino Acids; beta-Alanine metabolism | 303024.05 | 324898.87 | 0.351 |
| Metabolism; Glycan Biosynthesis and Metabolism; Glycosphingolipid biosynthesis - ganglio series | 15624.94 | 13467.47 | 0.349 |
| Metabolism; Lipid Metabolism; Lipid biosynthesis proteins | 615579.50 | 576482.04 | 0.337 |
| Metabolism; Xenobiotics Biodegradation and Metabolism; Drug metabolism - cytochrome P450 | 106037.63 | 122665.13 | 0.333 |
| Metabolism; Metabolism of Terpenoids and Polyketides; Geraniol degradation | 148626.13 | 179854.72 | 0.331 |
| Genetic Information Processing; Replication and Repair; Base excision repair | 379321.10 | 390167.07 | 0.331 |
| Metabolism; Metabolism of Terpenoids and Polyketides; Biosynthesis of type II polyketide backbone | 2.63 | 5.10 | 0.327 |
| Metabolism; Carbohydrate Metabolism; Pyruvate metabolism | 1227203.26 | 1191757.24 | 0.327 |
| Organismal Systems; Endocrine System; Insulin signaling pathway | 63122.71 | 64820.73 | 0.326 |
| Metabolism; Glycan Biosynthesis and Metabolism; Glycosaminoglycan degradation | 23453.89 | 20422.53 | 0.324 |
| Metabolism; Amino Acid Metabolism; Phenylalanine, tyrosine and tryptophan biosynthesis | 718085.53 | 694250.28 | 0.321 |
| Cellular Processes; Cell Growth and Death; p53 signaling pathway | 7789.40 | 4208.61 | 0.320 |
| Metabolism; Biosynthesis of Other Secondary Metabolites; Butirosin and neomycin biosynthesis | 46471.85 | 41137.70 | 0.317 |
| Human Diseases; Cancers; Small cell lung cancer | 7789.08 | 4150.66 | 0.317 |
| Human Diseases; Cancers; Colorectal cancer | 7789.08 | 4150.60 | 0.317 |
| Human Diseases; Cardiovascular Diseases; Viral myocarditis | 7789.08 | 4150.60 | 0.317 |
| Human Diseases; Infectious Diseases; Toxoplasmosis | 7789.08 | 4150.60 | 0.317 |
| Human Diseases; Infectious Diseases; Influenza A | 7795.35 | 4150.79 | 0.317 |
| Genetic Information Processing; Folding, Sorting and Degradation; Chaperones and folding catalysts | 850763.64 | 871800.26 | 0.315 |
| Metabolism; Xenobiotics Biodegradation and Metabolism; Caprolactam degradation | 107565.78 | 132574.36 | 0.315 |
| Organismal Systems; Environmental Adaptation; Circadian rhythm - plant | 3466.62 | 5368.89 | 0.314 |
| Cellular Processes; Transport and Catabolism; Peroxisome | 203942.43 | 190022.50 | 0.313 |
| Human Diseases; Infectious Diseases; Epithelial cell signaling in Helicobacter pylori infection | 61873.48 | 57361.40 | 0.310 |
| Organismal Systems; Endocrine System; Melanogenesis | 12.25 | 6.81 | 0.310 |
| Cellular Processes; Cell Growth and Death; Apoptosis | 8202.38 | 4420.51 | 0.310 |
| Unclassified; Cellular Processes and Signaling; Sporulation | 271356.48 | 234343.12 | 0.302 |
| Metabolism; Amino Acid Metabolism; Histidine metabolism | 523966.82 | 503249.76 | 0.294 |
| Metabolism; Glycan Biosynthesis and Metabolism; Lipopolysaccharide biosynthesis proteins | 447692.68 | 516715.32 | 0.291 |
| Metabolism; Carbohydrate Metabolism; Galactose metabolism | 413481.06 | 448096.13 | 0.291 |
| Metabolism; Xenobiotics Biodegradation and Metabolism; Aminobenzoate degradation | 216455.48 | 262387.52 | 0.286 |
| Human Diseases; Cancers; Prostate cancer | 31447.88 | 29239.54 | 0.284 |
| Organismal Systems; Endocrine System; Progesterone-mediated oocyte maturation | 31447.88 | 29239.54 | 0.284 |
| Organismal Systems; Immune System; Antigen processing and presentation | 31447.88 | 29239.54 | 0.284 |
| Organismal Systems; Digestive System; Mineral absorption | 4109.65 | 6757.79 | 0.283 |
| Metabolism; Lipid Metabolism; Steroid biosynthesis | 8509.52 | 4424.44 | 0.281 |
| Metabolism; Lipid Metabolism; Synthesis and degradation of ketone bodies | 101349.51 | 86755.07 | 0.273 |
| Genetic Information Processing; Folding, Sorting and Degradation; Sulfur relay system | 414640.69 | 390356.44 | 0.272 |
| Metabolism; Carbohydrate Metabolism; Butanoate metabolism | 1031606.05 | 981126.64 | 0.270 |
| Organismal Systems; Endocrine System; PPAR signaling pathway | 132734.72 | 115953.30 | 0.270 |
| Unclassified; Genetic Information Processing; Restriction enzyme | 187740.87 | 153026.01 | 0.268 |
| Human Diseases; Infectious Diseases; Vibrio cholerae pathogenic cycle | 206059.05 | 154307.70 | 0.268 |
| Metabolism; Amino Acid Metabolism; Tryptophan metabolism | 325671.17 | 399174.15 | 0.268 |
| Organismal Systems; Endocrine System; Adipocytokine signaling pathway | 63209.11 | 54679.32 | 0.267 |
| Metabolism; Metabolism of Cofactors and Vitamins; Lipoic acid metabolism | 49324.43 | 54331.45 | 0.267 |
| Organismal Systems; Immune System; NOD-like receptor signaling pathway | 35528.06 | 31713.17 | 0.263 |
| Metabolism; Carbohydrate Metabolism; Propanoate metabolism | 780857.65 | 736074.71 | 0.254 |
| Metabolism; Metabolism of Other Amino Acids; D-Glutamine and D-glutamate metabolism | 141296.76 | 131122.89 | 0.254 |
| Metabolism; Biosynthesis of Other Secondary Metabolites; Caffeine metabolism | 828.93 | 369.23 | 0.254 |
| Metabolism; Xenobiotics Biodegradation and Metabolism; Naphthalene degradation | 239693.33 | 216508.76 | 0.252 |
| Unclassified; Cellular Processes and Signaling; Other transporters | 231814.79 | 245380.50 | 0.250 |
| Metabolism; Energy Metabolism; Photosynthesis proteins | 370955.68 | 322399.74 | 0.249 |
| Human Diseases; Cancers; Renal cell carcinoma | 11710.03 | 16497.01 | 0.249 |
| Metabolism; Metabolism of Cofactors and Vitamins; Biotin metabolism | 151491.32 | 133332.44 | 0.249 |
| Metabolism; Metabolism of Other Amino Acids; Phosphonate and phosphinate metabolism | 81890.42 | 74262.68 | 0.245 |
| Metabolism; Lipid Metabolism; Glycerolipid metabolism | 488873.04 | 436500.02 | 0.241 |
| Metabolism; Metabolism of Terpenoids and Polyketides; Prenyltransferases | 296557.44 | 269595.08 | 0.240 |
| Genetic Information Processing; Folding, Sorting and Degradation; Protein export | 528314.69 | 496127.33 | 0.240 |
| Metabolism; Lipid Metabolism; Ether lipid metabolism | 14198.09 | 9538.23 | 0.238 |
| Metabolism; Lipid Metabolism; Sphingolipid metabolism | 88215.26 | 63559.07 | 0.238 |
| Metabolism; Glycan Biosynthesis and Metabolism; Peptidoglycan biosynthesis | 614198.29 | 602680.62 | 0.230 |
| Environmental Information Processing; Membrane Transport; Secretion system | 1631675.89 | 1804699.38 | 0.230 |
| Genetic Information Processing; Translation; Ribosome biogenesis in eukaryotes | 41906.43 | 44233.10 | 0.229 |
| Metabolism; Lipid Metabolism; Biosynthesis of unsaturated fatty acids | 200390.95 | 224140.32 | 0.228 |
| Metabolism; Glycan Biosynthesis and Metabolism; Glycosphingolipid biosynthesis - globo series | 45953.81 | 37308.61 | 0.228 |
| Unclassified; Cellular Processes and Signaling; Other ion-coupled transporters | 1295970.55 | 1469732.78 | 0.228 |
| Metabolism; Energy Metabolism; Oxidative phosphorylation | 1120959.67 | 995507.16 | 0.224 |
| Metabolism; Energy Metabolism; Photosynthesis | 339759.98 | 298414.82 | 0.224 |
| Human Diseases; Neurodegenerative Diseases; Parkinson's disease | 28538.85 | 19312.94 | 0.222 |
| Genetic Information Processing; Folding, Sorting and Degradation; Protein processing in endoplasmic reticulum | 61473.94 | 53866.81 | 0.221 |
| Metabolism; Metabolism of Terpenoids and Polyketides; Biosynthesis of ansamycins | 96178.44 | 110001.97 | 0.218 |
| Metabolism; Amino Acid Metabolism; Glycine, serine and threonine metabolism | 904224.24 | 885604.01 | 0.217 |
| Metabolism; Glycan Biosynthesis and Metabolism; N-Glycan biosynthesis | 37086.93 | 23676.76 | 0.215 |
| Metabolism; Lipid Metabolism; Fatty acid elongation in mitochondria | 70.21 | 25.49 | 0.214 |
| Metabolism; Xenobiotics Biodegradation and Metabolism; Fluorobenzoate degradation | 16529.77 | 30079.92 | 0.210 |
| Genetic Information Processing; Transcription; Basal transcription factors | 2464.99 | 4674.65 | 0.209 |
| Human Diseases; Neurodegenerative Diseases; Alzheimer's disease | 73976.63 | 65533.35 | 0.203 |
| Organismal Systems; Immune System; RIG-I-like receptor signaling pathway | 2337.60 | 4307.92 | 0.200 |
| Metabolism; Xenobiotics Biodegradation and Metabolism; Nitrotoluene degradation | 133590.15 | 109259.65 | 0.196 |
| Metabolism; Xenobiotics Biodegradation and Metabolism; Bisphenol degradation | 87968.88 | 67213.35 | 0.196 |
| Environmental Information Processing; Signal Transduction; Notch signaling pathway | 640.80 | 207.56 | 0.196 |
| Environmental Information Processing; Signal Transduction; Wnt signaling pathway | 640.80 | 207.56 | 0.196 |
| Human Diseases; Cancers; Chronic myeloid leukemia | 640.80 | 207.56 | 0.196 |
| Metabolism; Biosynthesis of Other Secondary Metabolites; Isoflavonoid biosynthesis | 16.97 | 78.98 | 0.191 |
| Unclassified; Genetic Information Processing; Transcription related proteins | 17755.04 | 26407.74 | 0.191 |
| Metabolism; Lipid Metabolism; Glycerophospholipid metabolism | 541862.46 | 517980.73 | 0.191 |
| Genetic Information Processing; Translation; mRNA surveillance pathway | 58.12 | 637.72 | 0.190 |
| Metabolism; Metabolism of Cofactors and Vitamins; Folate biosynthesis | 426127.75 | 392270.99 | 0.189 |
| Human Diseases; Infectious Diseases; Staphylococcus aureus infection | 15876.18 | 9905.68 | 0.188 |
| Environmental Information Processing; Signal Transduction; VEGF signaling pathway | 0.00 | 0.06 | 0.187 |
| Human Diseases; Infectious Diseases; Leishmaniasis | 0.00 | 0.06 | 0.187 |
| Cellular Processes; Cell Communication; Focal adhesion | 0.00 | 0.45 | 0.187 |
| Environmental Information Processing; Signaling Molecules and Interaction; CAM ligands | 0.00 | 0.45 | 0.187 |
| Environmental Information Processing; Signaling Molecules and Interaction; ECM-receptor interaction | 0.00 | 0.45 | 0.187 |
| Metabolism; Amino Acid Metabolism; Tyrosine metabolism | 451355.63 | 483225.10 | 0.186 |
| Metabolism; Carbohydrate Metabolism; Amino sugar and nucleotide sugar metabolism | 1163731.96 | 1127592.53 | 0.185 |
| Environmental Information Processing; Signaling Molecules and Interaction; Neuroactive ligand-receptor interaction | 4.29 | 0.19 | 0.184 |
| Metabolism; Glycan Biosynthesis and Metabolism; Glycosphingolipid biosynthesis - lacto and neolacto series | 0.00 | 2.79 | 0.181 |
| Organismal Systems; Circulatory System; Cardiac muscle contraction | 20750.28 | 15161.57 | 0.180 |
| Metabolism; Biosynthesis of Other Secondary Metabolites; Streptomycin biosynthesis | 304376.65 | 271093.31 | 0.180 |
| Metabolism; Metabolism of Cofactors and Vitamins; Retinol metabolism | 61380.95 | 70376.11 | 0.180 |
| Metabolism; Carbohydrate Metabolism; C5-Branched dibasic acid metabolism | 326194.45 | 303530.57 | 0.178 |
| Metabolism; Metabolism of Terpenoids and Polyketides; Biosynthesis of 12-, 14- and 16-membered macrolides | 36.49 | 4.86 | 0.178 |
| Unclassified; Genetic Information Processing; Protein folding and associated processing | 756123.01 | 732869.20 | 0.177 |
| Metabolism; Energy Metabolism; Carbon fixation pathways in prokaryotes | 1201018.74 | 1058955.83 | 0.177 |
| Metabolism; Metabolism of Terpenoids and Polyketides; Polyketide sugar unit biosynthesis | 182319.22 | 153425.42 | 0.176 |
| Metabolism; Xenobiotics Biodegradation and Metabolism; Chloroalkane and chloroalkene degradation | 330535.60 | 284023.13 | 0.176 |
| Metabolism; Biosynthesis of Other Secondary Metabolites; Novobiocin biosynthesis | 150874.30 | 138993.11 | 0.174 |
| Metabolism; Metabolism of Terpenoids and Polyketides; Sesquiterpenoid biosynthesis | 8.90 | 0.32 | 0.174 |
| Human Diseases; Infectious Diseases; Vibrio cholerae infection | 33656.22 | 13251.06 | 0.171 |
| Metabolism; Biosynthesis of Other Secondary Metabolites; Flavonoid biosynthesis | 23992.08 | 16171.47 | 0.170 |
| Metabolism; Amino Acid Metabolism; Alanine, aspartate and glutamate metabolism | 1002940.09 | 909295.62 | 0.170 |
| Metabolism; Lipid Metabolism; Fatty acid biosynthesis | 494072.38 | 450066.17 | 0.169 |
| Metabolism; Nucleotide Metabolism; Pyrimidine metabolism | 1578161.45 | 1441064.22 | 0.166 |
| Unclassified; Cellular Processes and Signaling; Cell motility and secretion | 239629.55 | 218520.44 | 0.165 |
| Metabolism; Metabolism of Terpenoids and Polyketides; Biosynthesis of vancomycin group antibiotics | 53264.39 | 44233.38 | 0.164 |
| Metabolism; Carbohydrate Metabolism; Fructose and mannose metabolism | 761312.10 | 838949.34 | 0.164 |
| Metabolism; Metabolism of Terpenoids and Polyketides; Carotenoid biosynthesis | 44826.00 | 22225.41 | 0.163 |
| Metabolism; Biosynthesis of Other Secondary Metabolites; Betalain biosynthesis | 4843.92 | 433.12 | 0.161 |
| Metabolism; Metabolism of Terpenoids and Polyketides; Zeatin biosynthesis | 34580.80 | 32012.44 | 0.161 |
| Human Diseases; Cardiovascular Diseases; Hypertrophic cardiomyopathy (HCM) | 37.54 | 737.44 | 0.159 |
| Metabolism; Biosynthesis of Other Secondary Metabolites; Indole alkaloid biosynthesis | 4776.71 | 329.62 | 0.157 |
| Metabolism; Metabolism of Terpenoids and Polyketides; Biosynthesis of type II polyketide products | 4165.61 | 75.39 | 0.157 |
| Unclassified; Metabolism; Energy metabolism | 969829.47 | 885398.31 | 0.155 |
| Organismal Systems; Excretory System; Vasopressin-regulated water reabsorption | 2108.28 | 18.92 | 0.154 |
| Metabolism; Biosynthesis of Other Secondary Metabolites; Stilbenoid, diarylheptanoid and gingerol biosynthesis | 29575.20 | 17305.14 | 0.153 |
| Organismal Systems; Environmental Adaptation; Plant-pathogen interaction | 164957.46 | 141675.60 | 0.152 |
| Environmental Information Processing; Membrane Transport; Phosphotransferase system (PTS) | 476569.29 | 705383.31 | 0.148 |
| Metabolism; Energy Metabolism; Sulfur metabolism | 344978.33 | 310705.65 | 0.147 |
| Environmental Information Processing; Membrane Transport; Transporters | 6269641.44 | 7309438.31 | 0.146 |
| Metabolism; Carbohydrate Metabolism; Pentose phosphate pathway | 791998.76 | 838383.86 | 0.141 |
| Unclassified; Metabolism; Amino acid metabolism | 271314.53 | 295238.17 | 0.136 |
| Environmental Information Processing; Membrane Transport; ABC transporters | 3690748.81 | 4287538.75 | 0.135 |
| Metabolism; Glycan Biosynthesis and Metabolism; Glycosylphosphatidylinositol(GPI)-anchor biosynthesis | 2.12 | 0.00 | 0.135 |
| Metabolism; Xenobiotics Biodegradation and Metabolism; Styrene degradation | 58682.98 | 82028.07 | 0.134 |
| Human Diseases; Neurodegenerative Diseases; Amyotrophic lateral sclerosis (ALS) | 21830.66 | 34364.17 | 0.133 |
| Metabolism; Lipid Metabolism; Primary bile acid biosynthesis | 13287.87 | 8698.13 | 0.133 |
| Metabolism; Metabolism of Other Amino Acids; Selenocompound metabolism | 382143.49 | 365218.99 | 0.130 |
| Metabolism; Metabolism of Other Amino Acids; Glutathione metabolism | 328563.02 | 362933.16 | 0.128 |
| Unclassified; Cellular Processes and Signaling; Cell division | 54017.05 | 62196.90 | 0.128 |
| Genetic Information Processing; Transcription; RNA polymerase | 150166.78 | 128172.01 | 0.127 |
| Metabolism; Nucleotide Metabolism; Purine metabolism | 2098585.06 | 1933766.43 | 0.125 |
| Genetic Information Processing; Replication and Repair; DNA repair and recombination proteins | 2472918.89 | 2336209.56 | 0.124 |
| Cellular Processes; Cell Motility; Flagellar assembly | 437333.09 | 336079.97 | 0.123 |
| Unclassified; Metabolism; Lipid metabolism | 96706.91 | 130244.85 | 0.121 |
| Metabolism; Xenobiotics Biodegradation and Metabolism; Benzoate degradation | 333933.07 | 415859.86 | 0.120 |
| Unclassified; Genetic Information Processing; Translation proteins | 823984.46 | 763179.76 | 0.119 |
| Organismal Systems; Digestive System; Pancreatic secretion | 5.52 | 0.26 | 0.118 |
| Metabolism; Metabolism of Terpenoids and Polyketides; Terpenoid backbone biosynthesis | 491314.69 | 443574.86 | 0.116 |
| Human Diseases; Infectious Diseases; Chagas disease (American trypanosomiasis) | 9739.36 | 14628.14 | 0.114 |
| Metabolism; Lipid Metabolism; Secondary bile acid biosynthesis | 9331.65 | 7323.67 | 0.110 |
| Unclassified; Poorly Characterized; Function unknown | 1581314.67 | 1792827.87 | 0.109 |
| Metabolism; Amino Acid Metabolism; Valine, leucine and isoleucine biosynthesis | 714995.80 | 640138.70 | 0.108 |
| Metabolism; Glycan Biosynthesis and Metabolism; Various types of N-glycan biosynthesis | 67.38 | 166.43 | 0.108 |
| Metabolism; Amino Acid Metabolism; Phenylalanine metabolism | 254511.84 | 315110.90 | 0.103 |
| Unclassified; Cellular Processes and Signaling; Inorganic ion transport and metabolism | 252970.38 | 321770.23 | 0.103 |
| Unclassified; Metabolism; Glycan biosynthesis and metabolism | 54107.74 | 73110.90 | 0.103 |
| Human Diseases; Infectious Diseases; Shigellosis | 0.74 | 4.85 | 0.100 |
| Unclassified; Cellular Processes and Signaling; Membrane and intracellular structural molecules | 550382.26 | 642979.70 | 0.099 |
| Environmental Information Processing; Signaling Molecules and Interaction; G protein-coupled receptors | 44.89 | 1967.07 | 0.095 |
| Genetic Information Processing; Replication and Repair; Nucleotide excision repair | 311560.17 | 274964.94 | 0.094 |
| Metabolism; Carbohydrate Metabolism; Starch and sucrose metabolism | 734177.77 | 799791.12 | 0.092 |
| Environmental Information Processing; Signal Transduction; Phosphatidylinositol signaling system | 101617.42 | 90221.61 | 0.088 |
| Metabolism; Metabolism of Cofactors and Vitamins; Pantothenate and CoA biosynthesis | 582069.73 | 532122.93 | 0.087 |
| Genetic Information Processing; Transcription; Transcription factors | 1486082.05 | 1893316.25 | 0.086 |
| Environmental Information Processing; Membrane Transport; Bacterial secretion system | 726111.78 | 842094.68 | 0.084 |
| Human Diseases; Metabolic Diseases; Type I diabetes mellitus | 52476.37 | 42900.26 | 0.081 |
| Metabolism; Xenobiotics Biodegradation and Metabolism; Toluene degradation | 141021.10 | 160622.15 | 0.080 |
| Unclassified; Cellular Processes and Signaling; Pores ion channels | 389994.96 | 501113.01 | 0.080 |
| Genetic Information Processing; Transcription; Transcription machinery | 743481.03 | 615266.27 | 0.074 |
| Cellular Processes; Cell Motility; Bacterial motility proteins | 1277725.31 | 1037556.51 | 0.073 |
| Metabolism; Metabolism of Cofactors and Vitamins; Ubiquinone and other terpenoid-quinone biosynthesis | 245916.41 | 317879.68 | 0.073 |
| Human Diseases; Metabolic Diseases; Type II diabetes mellitus | 47543.05 | 43793.82 | 0.070 |
| Unclassified; Metabolism; Nucleotide metabolism | 37106.15 | 72598.65 | 0.069 |
| Genetic Information Processing; Replication and Repair; DNA replication proteins | 968959.07 | 887382.48 | 0.068 |
| Metabolism; Metabolism of Cofactors and Vitamins; Thiamine metabolism | 409880.73 | 385613.66 | 0.068 |
| Metabolism; Xenobiotics Biodegradation and Metabolism; Xylene degradation | 56072.40 | 67524.81 | 0.067 |
| Metabolism; Lipid Metabolism; Arachidonic acid metabolism | 49121.66 | 62641.80 | 0.066 |
| Metabolism; Xenobiotics Biodegradation and Metabolism; Ethylbenzene degradation | 73906.50 | 70762.15 | 0.064 |
| Genetic Information Processing; Folding, Sorting and Degradation; Proteasome | 36524.25 | 31897.57 | 0.062 |
| Genetic Information Processing; Folding, Sorting and Degradation; RNA degradation | 424462.07 | 385933.85 | 0.059 |
| Organismal Systems; Digestive System; Gastric acid secretion | 1.23 | 0.00 | 0.058 |
| Organismal Systems; Digestive System; Salivary secretion | 1.23 | 0.00 | 0.058 |
| Organismal Systems; Excretory System; Endocrine and other factor-regulated calcium reabsorption | 1.23 | 0.00 | 0.058 |
| Human Diseases; Infectious Diseases; African trypanosomiasis | 10106.13 | 16690.64 | 0.058 |
| Metabolism; Metabolism of Terpenoids and Polyketides; Tetracycline biosynthesis | 168231.87 | 136080.37 | 0.055 |
| Genetic Information Processing; Replication and Repair; DNA replication | 537366.19 | 484487.22 | 0.054 |
| Environmental Information Processing; Signaling Molecules and Interaction; Bacterial toxins | 135613.73 | 93385.53 | 0.054 |
| Human Diseases; Infectious Diseases; Tuberculosis | 160882.54 | 125665.39 | 0.053 |
| Cellular Processes; Cell Growth and Death; Cell cycle | 1.98 | 0.00 | 0.050 |
| Cellular Processes; Transport and Catabolism; Phagosome | 1.98 | 0.00 | 0.050 |
| Environmental Information Processing; Signal Transduction; mTOR signaling pathway | 1.98 | 0.00 | 0.050 |
| Human Diseases; Infectious Diseases; Hepatitis C | 1.98 | 0.00 | 0.050 |
| Human Diseases; Infectious Diseases; Measles | 1.98 | 0.00 | 0.050 |
| Metabolism; Metabolism of Other Amino Acids; Cyanoamino acid metabolism | 167940.56 | 240336.81 | 0.049 |
| Metabolism; Enzyme Families; Protein kinases | 354701.94 | 396159.58 | 0.049 |
| Metabolism; Xenobiotics Biodegradation and Metabolism; Dioxin degradation | 57975.22 | 86084.01 | 0.048 |
| Unclassified; Cellular Processes and Signaling; Germination | 7119.70 | 31478.87 | 0.048 |
| Environmental Information Processing; Signaling Molecules and Interaction; Cellular antigens | 44131.83 | 57618.60 | 0.047 |
| Metabolism; Metabolism of Cofactors and Vitamins; Riboflavin metabolism | 234991.73 | 264989.79 | 0.045 |
| Metabolism; Metabolism of Terpenoids and Polyketides; Biosynthesis of siderophore group nonribosomal peptides | 41068.70 | 102163.90 | 0.045 |
| Organismal Systems; Digestive System; Carbohydrate digestion and absorption | 7683.19 | 15838.50 | 0.043 |
| Cellular Processes; Cell Growth and Death; Cell cycle - Caulobacter | 456406.73 | 371992.82 | 0.043 |
| Human Diseases; Neurodegenerative Diseases; Prion diseases | 4929.08 | 12172.13 | 0.043 |
| Human Diseases; Infectious Diseases; Bacterial invasion of epithelial cells | 477.96 | 1886.74 | 0.042 |
| Organismal Systems; Endocrine System; Renin-angiotensin system | 223.14 | 3508.07 | 0.041 |
| Metabolism; Carbohydrate Metabolism; Inositol phosphate metabolism | 179659.25 | 211878.59 | 0.040 |
| Genetic Information Processing; Replication and Repair; Homologous recombination | 755669.44 | 689152.89 | 0.038 |
| Cellular Processes; Cell Motility; Bacterial chemotaxis | 594237.44 | 429275.50 | 0.038 |
| Unclassified; Metabolism; Carbohydrate metabolism | 113761.87 | 148281.53 | 0.038 |
| Metabolism; Lipid Metabolism; Linoleic acid metabolism | 65744.96 | 51634.82 | 0.036 |
| Human Diseases; Infectious Diseases; Pertussis | 17829.19 | 95162.96 | 0.034 |
| Metabolism; Energy Metabolism; Methane metabolism | 1119302.07 | 995824.42 | 0.032 |
| Genetic Information Processing; Translation; Ribosome | 1889043.17 | 1669820.68 | 0.031 |
| Metabolism; Glycan Biosynthesis and Metabolism; Glycosyltransferases | 322299.14 | 340979.95 | 0.028 |
| Metabolism; Biosynthesis of Other Secondary Metabolites; Phenylpropanoid biosynthesis | 71079.94 | 116445.70 | 0.026 |
| Metabolism; Carbohydrate Metabolism; Pentose and glucuronate interconversions | 449864.85 | 553534.67 | 0.026 |
| Metabolism; Carbohydrate Metabolism; Ascorbate and aldarate metabolism | 116571.99 | 225889.70 | 0.022 |
| Genetic Information Processing; Translation; Aminoacyl-tRNA biosynthesis | 1000939.76 | 868401.28 | 0.021 |
| Genetic Information Processing; Folding, Sorting and Degradation; Ubiquitin system | 10433.06 | 18806.87 | 0.021 |
| Unclassified; Metabolism; Biosynthesis and biodegradation of secondary metabolites | 56525.18 | 120175.94 | 0.021 |
| Metabolism; Metabolism of Cofactors and Vitamins; Porphyrin and chlorophyll metabolism | 1144422.97 | 910981.33 | 0.020 |
| Genetic Information Processing; Replication and Repair; Mismatch repair | 690125.95 | 605918.81 | 0.018 |
| Metabolism; Amino Acid Metabolism; Amino acid related enzymes | 1314959.13 | 1196690.05 | 0.017 |
| Unclassified; Poorly Characterized; General function prediction only | 3527647.35 | 3353651.70 | 0.016 |
| Unclassified; Cellular Processes and Signaling; Electron transfer carriers | 18352.14 | 55324.80 | 0.016 |
| Organismal Systems; Excretory System; Aldosterone-regulated sodium reabsorption | 1.77 | 0.00 | 0.016 |
| Genetic Information Processing; Translation; Translation factors | 442374.12 | 390364.01 | 0.009 |
| Metabolism; Lipid Metabolism; alpha-Linolenic acid metabolism | 18777.10 | 37510.48 | 0.009 |
| Human Diseases; Cancers; Bladder cancer | 6660.62 | 12571.02 | 0.006 |
| Organismal Systems; Digestive System; Bile secretion | 6509.98 | 3393.46 | 0.005 |
| Unclassified; Metabolism; Others | 915381.30 | 991367.12 | 0.003 |
| Metabolism; Metabolism of Cofactors and Vitamins; One carbon pool by folate | 479807.61 | 427489.43 | 0.003 |
| Metabolism; Amino Acid Metabolism; Lysine biosynthesis | 652396.73 | 604740.37 | 0.002 |
| Metabolism; Biosynthesis of Other Secondary Metabolites; Flavone and flavonol biosynthesis | 415.78 | 52.06 | 0.002 |
| Environmental Information Processing; Signal Transduction; MAPK signaling pathway - yeast | 37873.70 | 34065.28 | 0.001 |
| Cellular Processes; Cell Motility; Cytoskeleton proteins | 269902.53 | 215037.43 | 0.000 |
| Unclassified; Genetic Information Processing; Replication, recombination and repair proteins | 814044.23 | 698467.69 | 0.000 |
| Cellular Processes; Cell Communication; Adherens junction | 0.00 | 0.00 | 0.000 |
| Cellular Processes; Cell Communication; Tight junction | 0.00 | 0.00 | 0.000 |
| Cellular Processes; Cell Growth and Death; Cell cycle - yeast | 0.00 | 0.00 | 0.000 |
| Cellular Processes; Cell Growth and Death; Oocyte meiosis | 0.00 | 0.00 | 0.000 |
| Cellular Processes; Cell Motility; Regulation of actin cytoskeleton | 0.00 | 0.00 | 0.000 |
| Environmental Information Processing; Signal Transduction; ErbB signaling pathway | 0.00 | 0.00 | 0.000 |
| Environmental Information Processing; Signal Transduction; Hedgehog signaling pathway | 0.00 | 0.00 | 0.000 |
| Environmental Information Processing; Signal Transduction; MAPK signaling pathway | 0.00 | 0.00 | 0.000 |
| Environmental Information Processing; Signal Transduction; TGF-beta signaling pathway | 0.00 | 0.00 | 0.000 |
| Environmental Information Processing; Signaling Molecules and Interaction; Cytokine receptors | 0.00 | 0.00 | 0.000 |
| Environmental Information Processing; Signaling Molecules and Interaction; Cytokine-cytokine receptor interaction | 0.00 | 0.00 | 0.000 |
| Environmental Information Processing; Signaling Molecules and Interaction; Glycan bindng proteins | 0.00 | 0.00 | 0.000 |
| Environmental Information Processing; Signaling Molecules and Interaction; GTP-binding proteins | 0.00 | 0.00 | 0.000 |
| Genetic Information Processing; Transcription; Spliceosome | 0.00 | 0.00 | 0.000 |
| Human Diseases; Cancers; Glioma | 0.00 | 0.00 | 0.000 |
| Human Diseases; Cancers; Pancreatic cancer | 0.00 | 0.00 | 0.000 |
| Human Diseases; Cardiovascular Diseases; Arrhythmogenic right ventricular cardiomyopathy (ARVC) | 0.00 | 0.00 | 0.000 |
| Human Diseases; Cardiovascular Diseases; Dilated cardiomyopathy (DCM) | 0.00 | 0.00 | 0.000 |
| Human Diseases; Immune System Diseases; Rheumatoid arthritis | 0.00 | 0.00 | 0.000 |
| Human Diseases; Infectious Diseases; Pathogenic Escherichia coli infection | 0.00 | 0.00 | 0.000 |
| Metabolism; Glycan Biosynthesis and Metabolism; Glycosaminoglycan biosynthesis - chondroitin sulfate | 0.00 | 0.00 | 0.000 |
| Metabolism; Glycan Biosynthesis and Metabolism; Other types of O-glycan biosynthesis | 0.00 | 0.00 | 0.000 |
| Organismal Systems; Circulatory System; Vascular smooth muscle contraction | 0.00 | 0.00 | 0.000 |
| Organismal Systems; Digestive System; Fat digestion and absorption | 0.00 | 0.00 | 0.000 |
| Organismal Systems; Immune System; Complement and coagulation cascades | 0.00 | 0.00 | 0.000 |
| Organismal Systems; Immune System; Cytosolic DNA-sensing pathway | 0.00 | 0.00 | 0.000 |
| Organismal Systems; Immune System; Fc epsilon RI signaling pathway | 0.00 | 0.00 | 0.000 |
| Organismal Systems; Immune System; Leukocyte transendothelial migration | 0.00 | 0.00 | 0.000 |
| Organismal Systems; Nervous System; Cholinergic synapse | 0.00 | 0.00 | 0.000 |
| Organismal Systems; Nervous System; Long-term depression | 0.00 | 0.00 | 0.000 |
| Organismal Systems; Nervous System; Long-term potentiation | 0.00 | 0.00 | 0.000 |
| Organismal Systems; Nervous System; Neurotrophin signaling pathway | 0.00 | 0.00 | 0.000 |
| Organismal Systems; Sensory System; Olfactory transduction | 0.00 | 0.00 | 0.000 |
| Organismal Systems; Sensory System; Phototransduction | 0.00 | 0.00 | 0.000 |
| Organismal Systems; Sensory System; Phototransduction - fly | 0.00 | 0.00 | 0.000 |
